# Supplementary material for: Partial removal of visceral epididymal white adipose tissue in obese Ldlr-/-.Leiden mice impacts adipokine secretion, plasma free fatty acids, and improves cerebrovascular health
Source: PLoS One. 2025 Oct 17;20(10):e0333024. doi: 10.1371/journal.pone.0333024 (PMC12533877; doi:10.1371/journal.pone.0333024)
Supplement: S1 File — (PDF) [file pone.0333024.s001.pdf]

S1 File: Supplementary methods

1. Surgical procedures

1.1 Analgesia

The surgical procedures were adapted from Mulder *et al.*[1]. One day before surgery and during 10 days after surgery, analgesia was provided in drinking water (rimadyl (carprofen), 0.067 mg/ml during 7 days and then 0.034 mg/ml during 3 days). 10 minutes before the surgery, a mixture (4 µl/g body weight) of fentanyl (0.06 mg/kg) and midazolam (0.2 mg/kg) was administered subcutaneously. After surgery, one dose of rimadyl (5 µl/g body weight) was additionally injected subcutaneously. Lidocaine spray (4%) was also used for pain relief of the peritoneum.

1.2 Procedures

Mice were anaesthetized with isoflurane (2.5-3%). Eyes were protected from drying with eye ointment, the abdomen were shaved and disinfected with 70% alcohol solution. In the HFD+WATx group, after a mid-ventral abdominal incision, the artery supplying the eWAT pad was ligated with insoluble suture threat. The eWAT pads were carefully removed and weighed. To prevent damage to testicular function and blood supply, the superficial layer of eWAT covering the gonads was not removed. This resulted, on average, in a removal of 1.4 g of eWAT corresponding to an estimated removal of 70% of the eWAT pad. This calculation is based on the weight of the removed tissue and data on the weight of total eWAT depots from previous time course experiment using same mice and same HFD [2]. In the HFD+sham group, a similar mid-ventral incision was made, the eWAT pads were pulled out but were left intact and placed back inside the peritoneal cavity. For all the operated mice, the peritoneum was sutured using dissolvable suture threat, the skin was sutured and 2-3 wound clips were placed. The mice were administered subcutaneously with 1 ml of warm saline solution to protect against dehydration and mice were placed in a pre-heated cage until waking up. Wound clips were removed after ~7 days when the skin was fully healed. The mice were monitored during two weeks by daily checks of the body weight and the scoring of well-being signs. A humane endpoint was predefined to ensure that mice exhibiting signs of severe deterioration of the health status, such as severe unexpected weight loss accompanied by severe infected wounds, signs of general sickness or discomfort and stop of water and food intake, could be euthanized.

2. Brain MRI

At t=7 weeks (i.e. prior to surgery), t=12 weeks (i.e.1 month after surgery) and t=27 weeks (i.e. 4 months after surgery), brain MRI was performed using a 11.7T BioSpec Avance III small animal MR system (Bruker Biospin, Ettlingen, Germany) with Paravision 6.0.1 software (Bruker) as already extensively described [3–5]. Prior to MRI, mice were anesthetized with isoflurane (3.5% for induction, 1.8% for maintenance) in a 1:2 oxygen-medical air mixture. The head of the mice was placed in a stereotactic holder to limit motion and eye ointment was placed on the eye to prevent dehydration. Both the respiration rate (pneumatic cushion) and body temperature (rectal probe) were monitored with a monitoring system (Small Animal Instruments Inc, Stony Brook, NY, USA).

2.1 Hippocampus volume and cortical thickness

Cortical thickness and hippocampus volume were measured in T2-weighted coronal images using ImageJ (v1.53, National Institutes of Health, United States) and the mouse brain atlas of Franklin and Paxinos [6]. Thickness of the auditory cortex (AUC, bregma -2.46), motor cortex (MC, bregma 1.10), somatosensory cortex (SSC, bregma -0.94) and visual cortex (VC, bregma -2.46) were measured manually. For each region, the measures from left and right hemispheres were averaged. Hippocampus volume was manually measured on 6 consecutive slices to cover the entire hippocampus (bregma -0.94 to -3.40). The measurements of the 6 slices from both hemisphere were summed and multiplied by slice thickness (0.5 mm). The overall cortical thickness was finally calculated as the average of AUC, MC, SSC and VC cortical thickness.

2.2 White matter and grey matter integrity

Diffusion Tensor Imaging (DTI) was performed to assess grey and white matter integrity based on fractional anisotropy and mean diffusivity: fractional anisotropy in white matter provides indication on the degree of myelination and fiber density, whereas mean diffusivity in grey matter describes an inverse measure of membrane density [7]. These two parameters were measured in white matter areas (corpus callosum, fornix and optic tract) and grey matter areas (AUC, MC, SSC, VC, hippocampus and combined caudate nucleus, globus pallidus, putamen). Measures for left and right hemispheres were averaged for each region and were eventually expressed as the average fractional anisotropy and the average mean diffusivity in grey matter and white matter.

2.3 Cerebral blood flow (CBF)

CBF was assessed using an Arterial Spin Labeling sequence with flow-sensitive alternating inversion recovery (FAIR) method [8]. Arterial Spin Labeling was first performed under normal gas mix (1:2 oxygen - medical air). The Arterial Spin Labeling sequence was repeated after switching to pure oxygen to induce vasoconstriction. Cerebral vasoreactivity, defined as the ability of the cerebrovasculature to adapt to constrict or dilate, was calculated by subtracting the CBF measured under vasoconstrictive conditions from the CBF measured under normal gas mix, divided by the CBF measured under normal gas mix [8]. CBF under normal gas mix, CBF under vasoconstrictive conditions and vasoreactivity were measured in the cortex, hippocampus, and thalamus.

2.4 Functional connectivity

Resting-state functional MRI acquisition was used to assess functional connectivity between specific regions of interest implicated in multiple cognitive and motor processes: ventral and dorsal hippocampus, auditory, somatosensory, motor, and visual cortical regions. Functional connectivity was determined from blood oxygen-dependent (BOLD) time series using total correlation analyses [9]. Z-scores were obtained after R-to-Z transformations and used for statistical analysis.

3. Behavioral tests

3.1 Morris Water Maze (MWM)

At t=6 weeks (prior to surgery), a MWM test was performed to assess spatial learning and short-term memory (hippocampal-dependent task). A pool (108 cm diameter) was filled with water (~22-24°C) and made opaque with non-dairy creamer. Four different cues were placed on the walls surrounding the pool at the four cardinal points (south, east, north, west). A circular platform (8 cm diameter) was placed 1 cm below the surface of water in the center of the North-East (NE) quadrant of the pool. During a first 4-day acquisition phase (learning phase), the mice were trained to find the hidden platform: the mice performed 4 trials per day starting each trial from a different cardinal point. Between each trial, mice were placed in their home-cage for at least 1 hour. For each trial, mice were allowed to find the platform during a maximum time of 120 seconds. The trial was automatically stopped either after 120 seconds if a mouse did not find the platform or after staying 30 seconds on the platform. When the mice did not find the platform, they were manually held on the platform during 30 seconds allowing them to look at the surroundings. The latency and distance to find the platform as well as the average velocity was measured for each trial and averaged per day. At the end of the fourth day a probe test was conducted: the platform was removed from the pool and the mice were allowed to swim during 120 seconds starting from south. The cumulated time

spent in the platform zone (platform location or within 2 cm around the platform) was measured in addition to the number of crossings of this zone. All trials and probe tests were recorded and analyzed with Ethovision XT (v15, Noldus, Wageningen, the Netherlands). The total distance moved and average velocity were determined. Before surgery, no group differences were observed between the Chow and HFD groups (S3 Fig).

3.2 Novel Object Recognition Test (ORT)

At=11 weeks (i.e. 1 month after surgery), an ORT was performed to assess explorative behavior and short-term (recognition) memory. The mice were first placed in an empty transparent box (45x45x30 cm) during 2 minutes for habituation. The ORT was then repeated over three consecutive days: each day the mice performed one "familiarization" trial and one "test" trial. During the "familiarization" trial, two identical objects were placed at equidistance from the center of the box and the mice were allowed to explore during 2 minutes and were then placed back in their home-cage. During the "test" trial, one familiar object was placed back in the box with one novel object that was different in shape and appearance, and the mice were allowed to explore again during 2 minutes. The objects included calcium eggs, yellow plastic ice cream cones, bottles filled with sand and water glasses. The object type was randomized and the position of the novel object (left or right) was balanced across the trials. Over the three ORT days, the interval between the "familiarization" and "test"' trials was respectively 30 min, 1 hour and 2 hours. All trials were tracked with Ethovison XT. Exploration of an object was defined as a direct contact with the object or movement within 2 cm around the object and automatically scored with Ethovision. For the "test" trial of each day, two indexes were calculated:

$$1)\ \ discrimination\ index = \frac{(\text{exploration of novel object} - \text{exploration of familiar object})}{(\text{exploration of novel object} + \text{exploration of familiar object})}$$

A score between -1 and 1 is obtained, a positive score indicates that the mice spent more time exploring the novel object, a negative score indicates that the mice spent more time exploring the familiar object and a score of 0 indicates no preference.

$$2)\ \ recognition\ index = \frac{\text{exploration of novel object}}{(\text{exploration of novel object} + \text{exploration of familiar object})}$$

The recognition index indicates the percentage of time spent exploring the novel object relative to the total exploration time. Of note, mice that did not explore any of the objects during the "familiarization" trial were excluded for the corresponding "test" trial and mice that did not explore any of the objects during the "test" trial were allocated a 0 value for both indexes.

3.3Reverse MWM

At t=26 weeks (i.e. 4 months after surgery), a reverse MWM test was performed to assess spatial learning, short-term and long-term memory. First, the last probe of the MWM was repeated to assess whether the mice remembered the former platform location (Probe 1, target quadrant NE). Then a new 2-day acquisition phase was conducted after placing the platform this time in the South-West quadrant (SW). 4 trials were performed per day, starting each time from a different cardinal point. At the end of the second day, the platform was removed and a probe test (Probe 2, target quadrant SW) was performed. The cumulated time spent in the platform zone as well as the number of crossings of this zone were determined with Ethovision.

4. Brain (immuno)histopathology

Whole brains were collected at sacrifice and the left hemisphere was fixed with paraformaldehyde (4%) for 24 to 48 hours. The left hemispheres were transferred to PBS (0.1 M) containing 0.01% sodium azide and stored at 4°C. One night before cryosection, the left hemispheres were placed in 30% sucrose in 0.1 M PBS for cryoprotection. Free-floating coronal sections (30 µm, 8 series) were prepared with a sliding microtome (HM 450, Walldorf, Germany). Diaminobenzidine-nickel immunohistochemical staining were performed on the free-floating sections using the protocol described in previous experiments [10]. 4 free-floating sections series were stained: 1) one was stained for ionized calcium-binding adapter molecule 1 (IBA-1), a general marker for activated microglia, using polyclonal goat anti-IBA-1 primary antibody (1:4000, #ab5076, Abcam, UK, RRID: AB_2224402) to assess neuroinflammation; 2) one for glial fibrillary acidic protein (GFAP), a marker to detect astrocytes, using polyclonal rabbit-anti-GFAP primary antibody (1:80000; Z0334, DAKO, Santa Clara, California, USA), to assess astrogliosis; 3) one for glucose transporter 1 (GLUT-1), a protein expressed in the cerebral microvasculature, using polyclonal rabbit anti-GLUT-1 primary antibody (1:80000, #07-140, Millipore, Burlington, MA, USA, RRID: AB_11212210) to assess vascular integrity; and 4) one for doublecortin (DCX), a marker for newly formed neurons, using polyclonal rabbit anti-DCX (1:4000, #326003, Synaptic Systems, Göttigen, Germany, RRID: AB_2620067) to assess neurogenesis. As secondary antibodies, either biotin-conjugated donkey anti-rabbit or anti-goat antibodies were used (1:1500, #711-065-152 (RRID: AB_2340593) and #705-065-147 (RRID: AB_2340397), Jackson Immunoresearch, Cambridgeshire, UK). The stained sections were mounted on gelatin-coated slides. IBA-1 and GLUT-1-stained slides were scanned with Aperio Digital Pathology Slide Scanner AT2 (20x magnification, Leica, Amsterdam, The Netherlands) and images of GFAP-stained slides were captured at 5× magnification using a Axio Imager A2 (Zeiss Germany), equipped with an RGB camera (AxioCam ERc 5s, Zeiss, Germany). For IBA-1, GFAP and GLUT-1-stained slides, up to three non-overlapping cross-sections at bregma ~-1.94 were selected depending on the availability and quality of the tissues and their results were averaged per mouse. In more detail, in ImageJ, grey matter areas (cortex,

hippocampus, thalamus) and white matter areas (corpus callosum, fimbria, optic tract, external capsule, internal capsule) were manually drawn by two independent assessors based on the aforementioned atlas. The immuno-positive area and the number of positive particles were determined using an automated quantification including intensity-based threshold to separate positive staining from the background. DCX-positive cells were manually quantified in the dentate gyrus of the hippocampus in up to three consecutive cross-sections by two independent assessors and the counts were averaged per mouse.

References

1. Mulder P, Morrison MC, Wielinga PY, Van Duyvenvoorde W, Kooistra T, Kleemann R. Surgical removal of inflamed epididymal white adipose tissue attenuates the development of non-alcoholic steatohepatitis in obesity. Int J Obes. 2016;40: 675–684. doi:10.1038/ijo.2015.226

2. Gart E, van Duyvenvoorde W, Snabel JM, de Ruiter C, Attema J, Caspers MPM, et al. Translational characterization of the temporal dynamics of metabolic dysfunctions in liver, adipose tissue and the gut during diet-induced NASH development in Ldlr−/−.Leiden mice. Heliyon. 2023;9: e13985. doi:10.1016/j.heliyon.2023.e13985

3. Lohkamp KJ, van den Hoek AM, Solé-Guardia G, Lisovets M, Alves Hoffmann T, Velanaki K, et al. The Preventive Effect of Exercise and Oral Branched-Chain Amino Acid Supplementation on Obesity-Induced Brain Changes in Ldlr−/−.Leiden Mice. Nutrients. 2023. doi:10.3390/nu15071716

4. Tengeler AC, Gart E, Wiesmann M, Arnoldussen IAC, van Duyvenvoorde W, Hoogstad M, et al. Propionic acid and not caproic acid, attenuates nonalcoholic steatohepatitis and improves (cerebro) vascular functions in obese Ldlr−/−.Leiden mice. FASEB J. 2020;34: 9575–9593. doi:10.1096/fj.202000455R

5. Arnoldussen IAC, Morrison MC, Wiesmann M, van Diepen JA, Worms N, Voskuilen M, et al. Milk fat globule membrane attenuates high fat diet-induced neuropathological changes in obese Ldlr−/−.Leiden mice. Int J Obes. 2022;46: 342–349. doi:10.1038/s41366-021-00998-w

6. Paxinos G, Franklin KBJ. The Mouse Brain in Stereotaxic Coordinates. 3rd ed. Academic Press: Cambridge;

7. Alexander AL, Lee JE, Lazar M, Field AS. Diffusion Tensor Imaging of the Brain. Neurotherapeutics. 2007;4: 316–329. doi:10.1016/j.nurt.2007.05.011

8. Wiesmann M, Zerbi V, Jansen D, Lütjohann D, Veltien A, Heerschap A, et al. Hypertension, cerebrovascular impairment, and cognitive decline in aged AβPP/PS1 mice. Theranostics. 2017;7: 1277–1289. doi:10.7150/thno.18509

9. Zerbi V, Wiesmann M, Emmerzaal TL, Jansen D, Beek M Van, Mutsaers MPC, et al. Resting-state functional connectivity changes in aging apoe4 and apoe-ko mice. J Neurosci. 2014;34: 13963–13975. doi:10.1523/JNEUROSCI.0684-14.2014

10. Janssen CIF, Zerbi V, Mutsaers MPC, de Jong BSW, Wiesmann M, Arnoldussen IAC, et al. Impact of dietary n-3 polyunsaturated fatty acids on cognition, motor skills and hippocampal neurogenesis in developing C57BL/6J mice. J Nutr Biochem. 2015;26: 24–35. doi:10.1016/j.jnutbio.2014.08.002
