# Supplementary material for: Partial removal of visceral epididymal white adipose tissue in obese Ldlr-/-.Leiden mice impacts adipokine secretion, plasma free fatty acids, and improves cerebrovascular health
Source: PLoS One. 2025 Oct 17;20(10):e0333024. doi: 10.1371/journal.pone.0333024 (PMC12533877; doi:10.1371/journal.pone.0333024)
Supplement: S2 File — (PDF) [file pone.0333024.s002.pdf]

S2 File: Supplementary figures

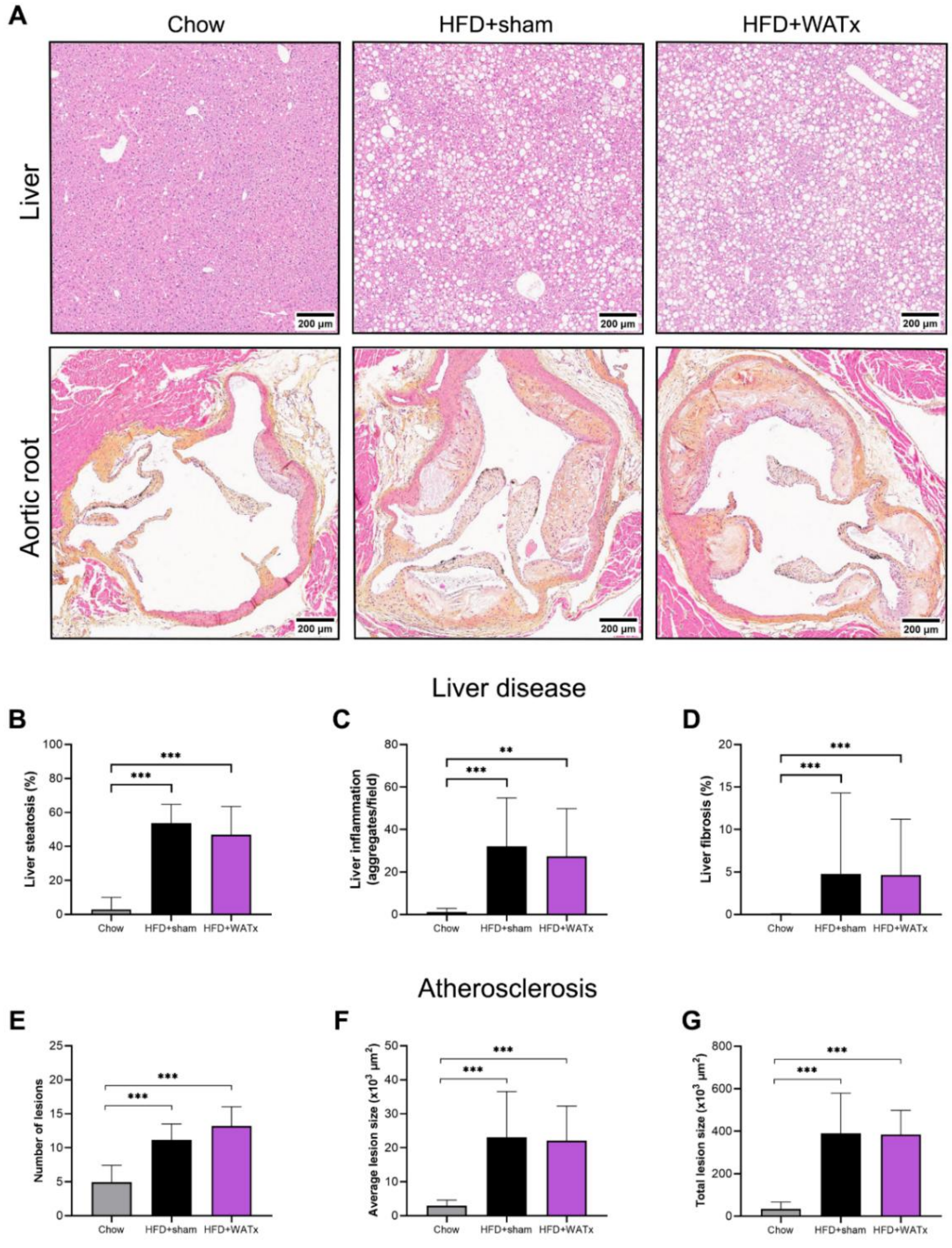

S1 Fig: Liver disease and atherosclerosis at the end of the study. (A) Representative pictures of liver and aortic root cross-sections respectively stained with hematoxylin-eosin (HE) and hematoxylin-phloxine-saffron (HPS). (B) Liver steatosis and (C) lobular inflammation were quantified in HE-stained liver cross-sections at t=28 weeks, and (D) liver fibrosis was measured as the percentage of Sirius red-positive area. Quantification of (E) The number of atherosclerotic lesions, (F) average lesion size and (G) total lesion size

were quantified in HPS-stained cross-sections of aortic roots at t=28 weeks. Data are shown as mean ± SD. ** p ≤ 0.01, *** p ≤ 0.001.

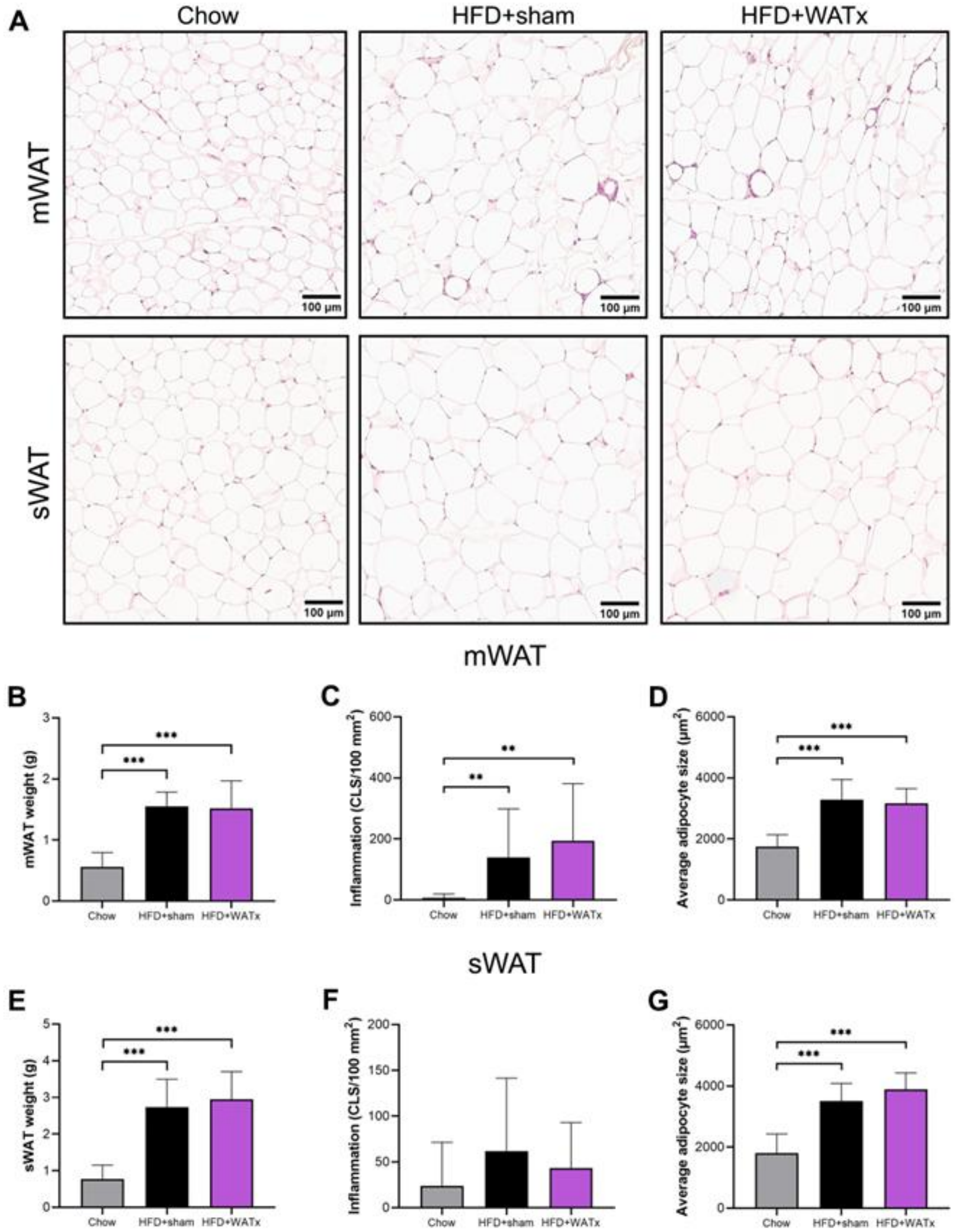

S2 Fig: Histological analyses of mesenteric (mWAT) and subcutaneous (sWAT) WAT at the end of the study. (A) Representative pictures of mWAT and sWAT histology at t=28 weeks. WAT weight, inflammation (crown-like structures, CLS) and adipocyte size were analyzed in (B-D) mWAT (inguinal) and (E-G) sWAT. Data are shown as mean ± SD. ** p≤0.01, *** p ≤ 0.001.

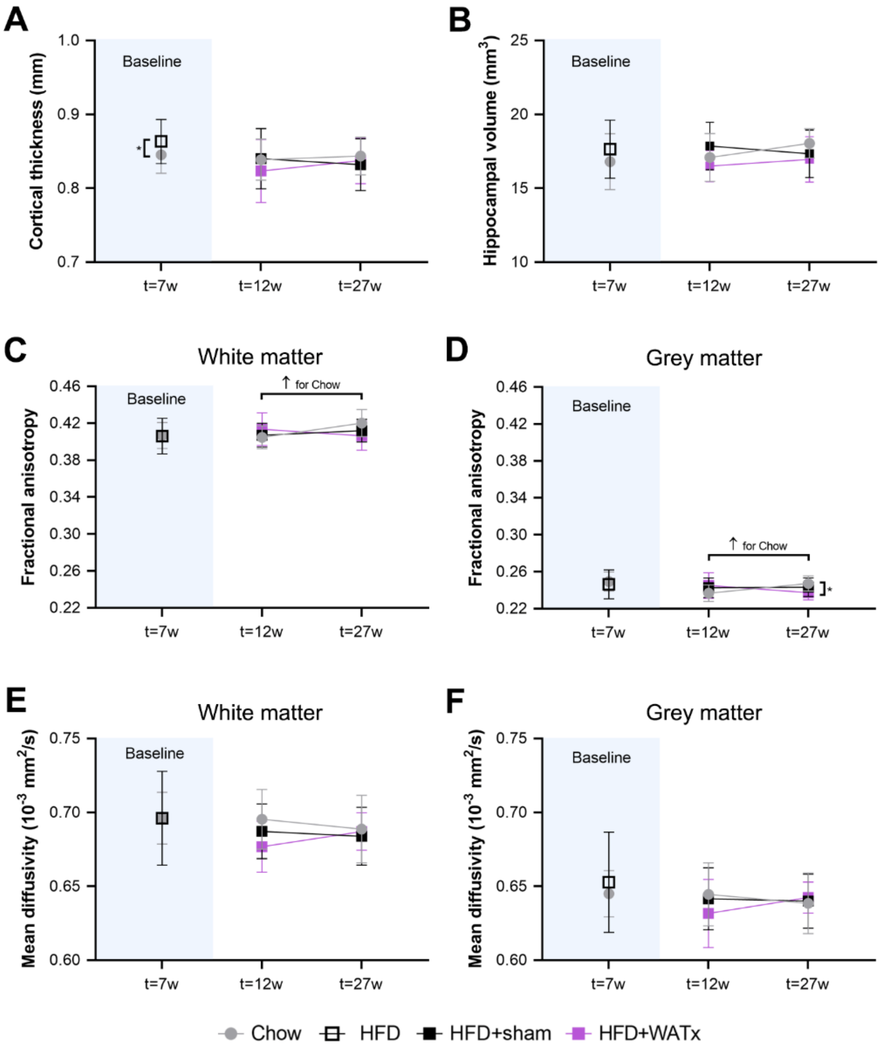

S3 Fig: Grey and white matter integrity. (A) Cortical thickness and (B) hippocampal volume measured in MRI anatomical images at t=7 weeks (prior to surgery), t=12 weeks (1 month after surgery), and t=27 weeks (4 months after surgery). (C-D) Fractional anisotropy and (E-F) mean diffusivity in grey and white matter based on DTI at the same times points. Data are shown as mean ± SD. ↑ increase over time for Chow group ($p \leq 0.05$). * $p \leq 0.05$ between the experimental groups.

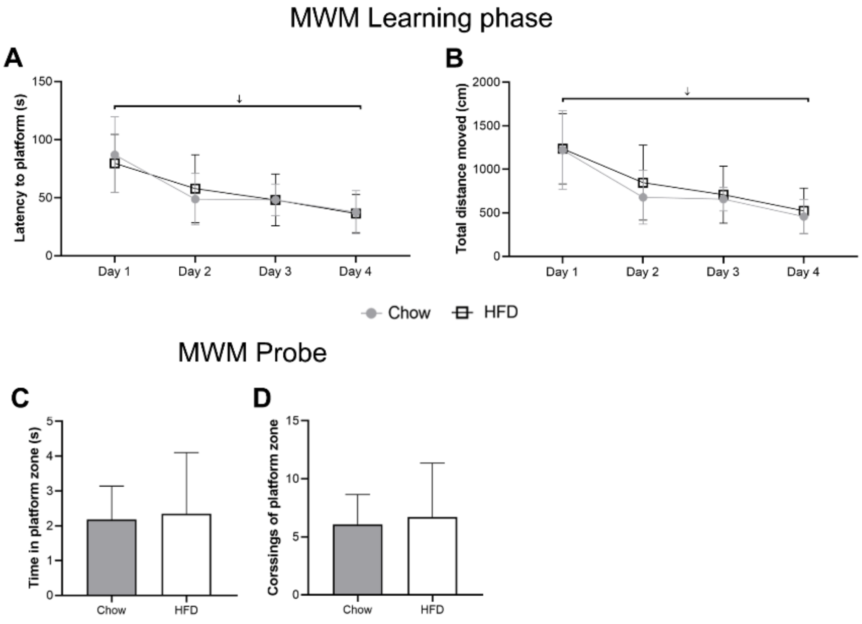

S4 Fig: Spatial learning and (short-term) memory performance before surgery. At t=6 weeks (prior to surgery), a Morris Water Maze (MWM) test was performed to assess (A-B) spatial learning (learning phase, 4 days) and (C-D) short-term memory (Probe). Data are shown as mean ± SD. ↓ decrease over time in both groups ($p \leq 0.05$).

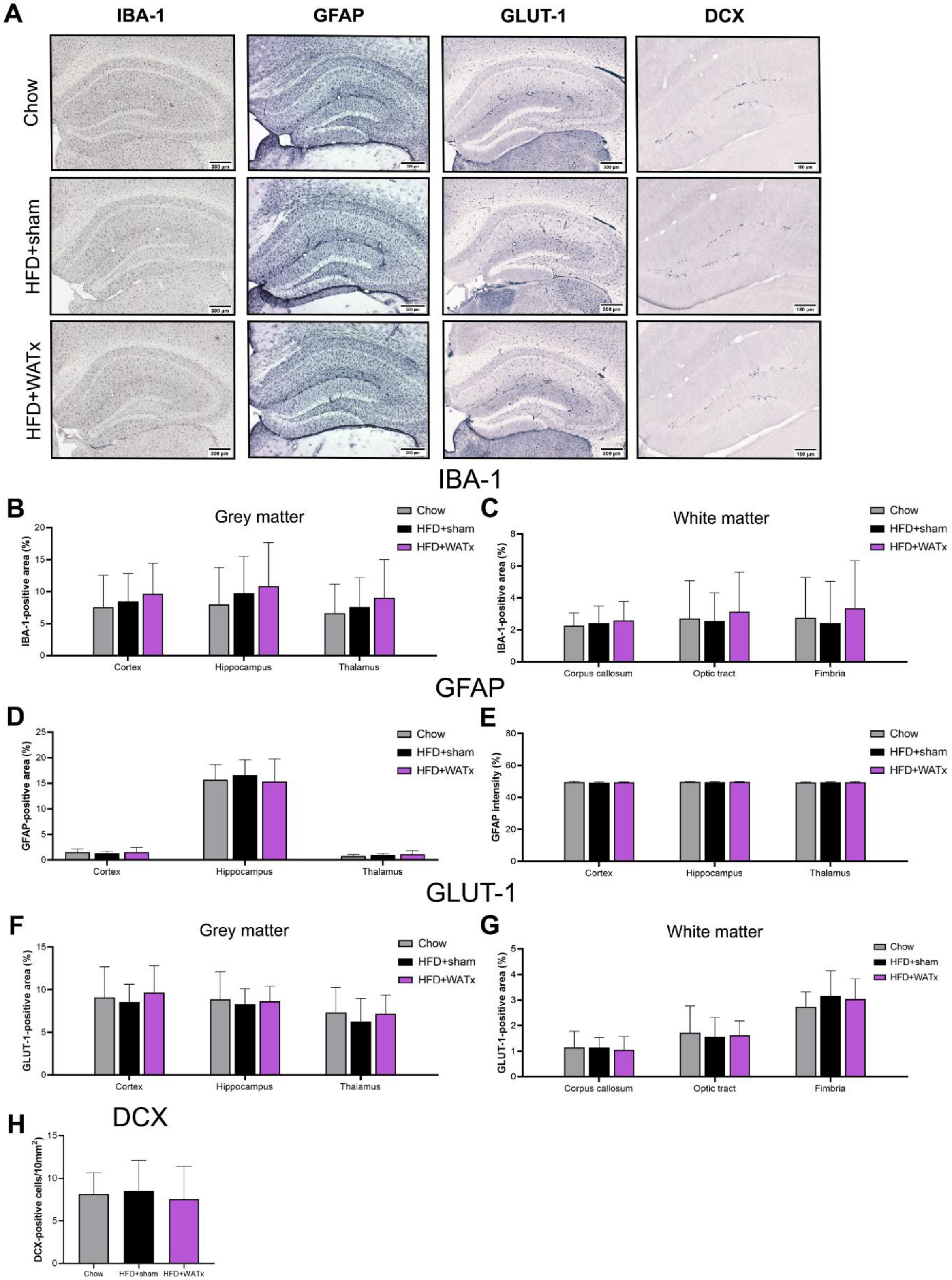

S5 Fig: Histological markers of neuroinflammation, vascular integrity and neurogenesis at the end of the study. Pathology of brain tissue was analyzed immunohistochemically at t=28 weeks. (A) Representative pictures. Quantification of (B-C) IBA-1-positive area as a marker for microglia activation

in grey and white matter regions, (D) GFAP-positive area and (E) intensity as a marker of astrogliosis, and (F-G) GLUT-1-positive area as an indicator of vascular integrity. (H) Neurogenesis was assessed by quantification of DCX-positive neurons in the dentate gyrus of the hippocampus. Data are shown as mean ± SD. Abbreviations: (DCX) doublecortin; (GFAP) glial fibrillary acidic protein; (GLUT-1) glucose transporter 1, (IBA-1) ionized calcium-binding adapter molecule 1.

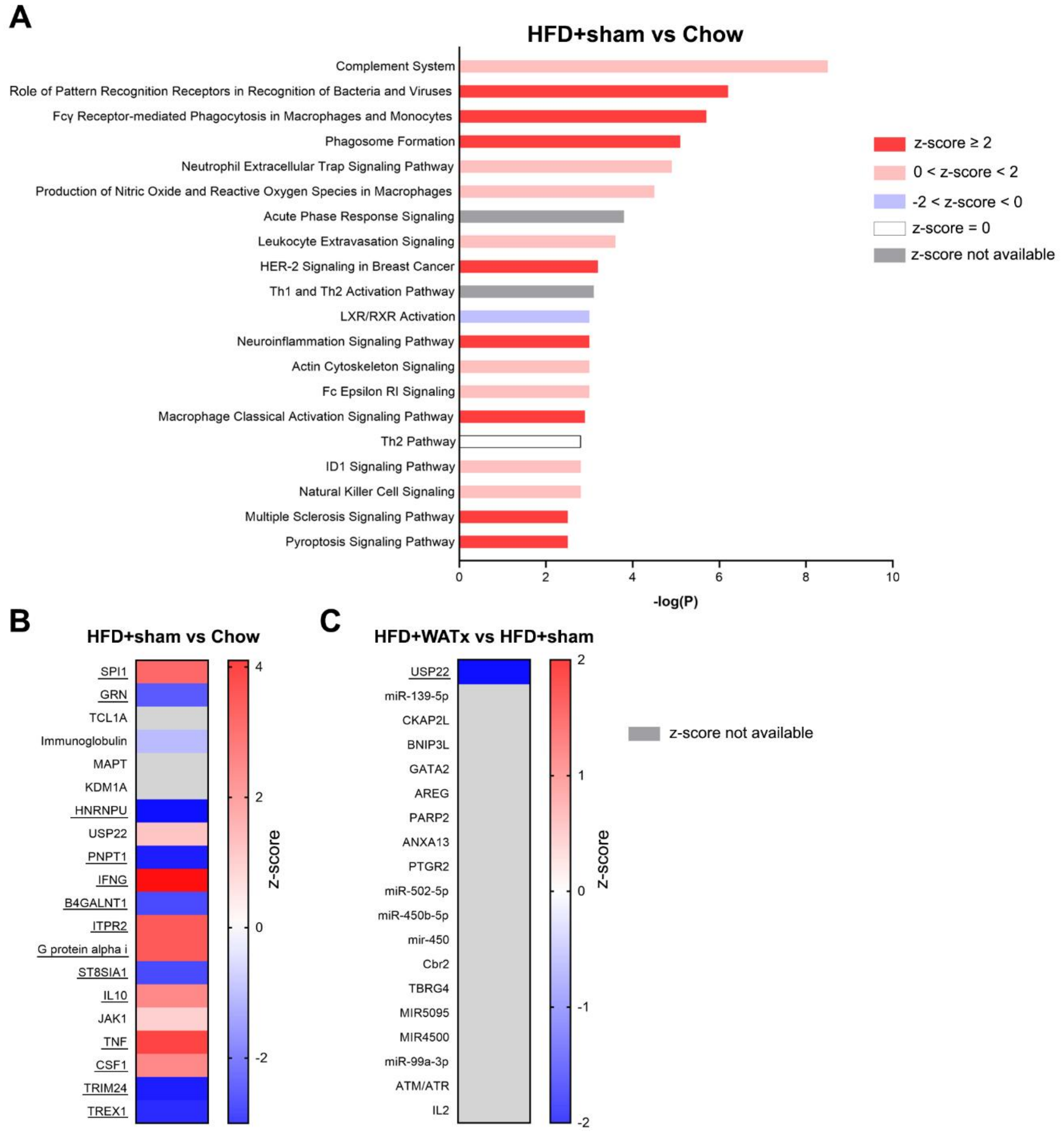

S6 Fig: Activation of canonical pathways and upstream regulators based on hippocampal gene expression. RNAseq in the hippocampus was performed at t=28 weeks (4 months after surgery). (A) 20 most significantly enriched canonical pathways based on hippocampus gene expression in HFD+sham vs Chow mice. No canonical pathways were enriched in HFD+WATx vs HFD+sham mice. Top 20 predicted upstream regulators based on hippocampus gene expression in (B) HFD+sham vs Chow mice and (C) HFD+WATx vs HFD+sham mice. The z-score indicates the predicted activation of a canonical pathway or upstream regulator: underlined z-scores correspond to either z-score ≤ -2 for relevant inhibition of the pathway or regulator (indicated in dark blue) or z-score ≥ 2 for relevant activation of the pathways or regulator (indicated in dark red). Upstream regulators are underlined when -log(p) < 2.
