# Supplementary material for: Partial removal of visceral epididymal white adipose tissue in obese Ldlr-/-.Leiden mice impacts adipokine secretion, plasma free fatty acids, and improves cerebrovascular health
Source: PLoS One. 2025 Oct 17;20(10):e0333024. doi: 10.1371/journal.pone.0333024 (PMC12533877; doi:10.1371/journal.pone.0333024)
Supplement: S3 File — (PDF) [file pone.0333024.s003.pdf]

S3 File: Supplementary tables

S1 Table: Sample size per experiment.

	t=5-7 weeks		t=8 weeks	t=12-14 weeks			t=26-28 weeks		
	Chow	HFD	HFD+WATx	Chow	HFD+sham	HFD+WATx	Chow	HFD+sham	HFD+WATx
**General measurements**									
BW	15	34	n/a	15	16[b]	12[b,e]	15	16[b]	11[c,d,e]
Blood glucose	15	34	n/a	15	15[b,f]	10[b,e,f]	14[a]	16[b]	11[c,d,e]
Plasma cholesterol	15	31[f]	n/a	15	16[b]	11[b,e,f]	14[a]	16[b]	11[c,d,e]
Plasma TG	15	31[f]	n/a	15	16[b]	12[b,e]	10[a,f]	16[b]	10[c,d,e,f]
Insulin	15	34	n/a	15	16[b]	12[b,e]	14[a]	16[b]	11[c,d,e]
**SBP**	12[g]	22[g]	n/a	n/a	n/a	n/a	15	11[b,g]	8[c,d,e,g]
**Behavioral tests**									
MWM	15	34	n/a	n/a	n/a	n/a	n/a	n/a	n/a
ORT	n/a	n/a	n/a	15	16[b]	12[b]	n/a	n/a	n/a
reverse MWM	n/a	n/a	n/a	n/a	n/a	n/a	15	16[b]	11[c,d,e]
**Brain MRI**									
Anatomical scan	15	34	n/a	15	16[b]	12[b,e]	14[a]	16[b]	11[c,d,e]
ASL	15	34	n/a	14[h]	16[b]	12[b,e]	14[a]	16[b]	11[c,d,e]
DTI	15	31-34[h]	n/a	15	16[b]	12[b,e]	14[a]	16[b]	11[c,d,e]
rsfMRI	15	34	n/a	15	16[b]	12[b,e]	14[a]	16[b]	11[c,d,e]
**Liver histology**	n/a	n/a	n/a	n/a	n/a	n/a	14[a]	16[b]	11[c,d,e]
**Atherosclerosis**	n/a	n/a	n/a	n/a	n/a	n/a	13[a,i]	14[b,i]	11[c,d,e]
**WAT histology**									
eWAT	n/a	n/a	15[c]	n/a	n/a	n/a	14[a]	16[b]	11[c,d,e]
sWAT	n/a	n/a	n/a	n/a	n/a	n/a	13[a,j]	16[b]	11[c,d,e]
mWAT	n/a	n/a	n/a	n/a	n/a	n/a	10[a,j]	16[b]	11[c,d,e]
**Brain histology**									
IBA-1	n/a	n/a	n/a	n/a	n/a	n/a	14[a]	16[b]	11[c,d,e]
GFAP	n/a	n/a	n/a	n/a	n/a	n/a	13[a,l]	16[b]	11[c,d,e]
GLUT-1	n/a	n/a	n/a	n/a	n/a	n/a	14[a]	16[b]	10[c,d,e,k]
DCX	n/a	n/a	n/a	n/a	n/a	n/a	13[a,i]	12[b,i]	9[c,d,e,i]
**Plasma markers**	n/a	n/a	n/a	n/a	n/a	n/a	14[a]	16[b]	11[c,d,e]
**Cortical markers**	n/a	n/a	n/a	n/a	n/a	n/a	14[a]	16[b]	11[c,d,e]
**WAT secretomes**									
eWAT	n/a	n/a	9[m]	n/a	n/a	n/a	7[m]	7[j,m]	5[j,m]
sWAT	n/a	n/a	n/a	n/a	n/a	n/a	7[m]	7[j,m]	5[j,m]
mWAT	n/a	n/a	n/a	n/a	n/a	n/a	4[j,m]	7[j,m]	4[j,m]
**HIP RNAseq**	n/a	n/a	n/a	n/a	n/a	n/a	12	16[b]	11[c,d,e]

[a] n=1 mouse died during the last MRI session; [b] n=1 mouse terminated earlier in the study due to surgery complications; [c] n=2 mice terminated earlier in the study due to surgery complications were excluded; [d] n=1 mouse terminated earlier in the study due to fighting-related wounds was excluded; [e] n=3 mice excluded because atrophied testes; [f] statistical outliers; [g] animals with < 3 valid measurements were excluded; [h] animal(s) or brain regions with motion artifacts had to be excluded; [i] animals with insufficient (< 12) aortic segments had to be excluded (aortas not perpendicular to cross-sectional plane); [j] tissues too small or of bad quality were excluded; [k] n=1 mouse not analyzed due to technical issues; [l] no cross-sections located at the right bregma or bad tissue quality; [m] half of the animals were analyzed. Abbreviations: (ASL) arterial spin labeling, (DTI) Diffusion Tensor Imaging, (DCX) doublecortin, (eWAT) epididymal WAT, (GLUT-1) glucose transporter 1, (HIP) hippocampus, (IBA-1) ionized calcium-binding adapter molecule 1, (MWM) Morris Water Maze, (mWAT) mesenteric WAT, (ORT) Object Recognition Test, (rsfMRI) resting-state functional MRI, (SBP) systolic blood pressure, (sWAT) subcutaneous WAT, (TG) triglycerides, (WAT) white adipose tissue.

S2 Table: Inflammatory proteins secreted within 4 hours by mWAT depots at the end of the study.

**Cytokines/chemokines**	**Secretion capacity of mWAT depot**		
	Chow	HFD+sham	HFD+WATx
**Adiponectin *(ng)***	**1065.8 ± 389.5[a]**	**1863.9 ± 252.0[b]**	**1630.6 ± 455.0[b]**
Resistin *(ng)*	148.2 ± 59.1	101.1 ± 58.1	99.7 ± 48.4
KC/CXCL1 *(pg)*	155021.2 ± 66776.2	200146.2 ± 165678.4	306516.8 ± 233316
**Leptin *(ng)***	**24.9 ± 29.2[a]**	**73.1 ± 39.9[b]**	**84.2 ± 54.2[b]**
MCP-1/CCL2 (*ng*)	15.6 ± 6.3	19.9 ± 16.7	17.5 ± 17.2
IP-10/CXCL10 *(pg)*	10085.6 ± 6843.7	6016.8 ± 4988.8	8761.8 ± 4353.8
PAI-1 (*pg*)	6822.0 ± 5025.1	6167.0 ± 4306.5	4878.9 ± 1546.6
IL-10 *(pg)*	922.7 ± 540.5	850.4 ± 803.8	2098.4 ± 2158.4
MIP-1α/CCL3 *(pg)*	285.8 ± 183.2	471.3 ± 682.4	433.9 ± 475.2
TNF-α *(pg)*	226.6 ± 113.5	152.3 ± 118.3	473.5 ± 470.2
RANTES/CCL5 *(pg)*	275.9 ± 191.6	268.5 ± 130.5	497.1 ± 228.0
IL-17 *(pg)*	n.d.	n.d.	n.d.

Concentrations of adipokines and inflammatory factors measured in culture media after 4 hours of ex-vivo culture of the mWAT depots collected at the end of the study (t=28 weeks). IL-17 data were excluded as most values were below the detection level (n.d.). Data for all other factors are shown as mean ± SD. Statistical differences are indicated in bold; groups with the same superscript letters are statistically comparable ($p>0.05$) and groups with different superscript letters are statistically different ($p\leq0.05$). Abbreviations: (IL-10) interleukin 10; (IL-17) interleukin 17; (IP-10/CXCL10) interferon ɣ-inducible protein 10; (KC/CXCL1) keratinocyte chemoattractant; (MCP-1/CCL2) monocyte chemoattractant protein 1; (MIP-1α/CCL3) macrophage inflammatory protein 1α; (PAI-1) plasminogen activator inhibitor 1; (RANTES/CCL5) regulated on activation, normal T-cell expressed and secreted; (TNF-α) tumor necrosis factor α.

S3 Table: Inflammatory proteins secreted within 4 hours by sWAT depots at the end of the study.

**Cytokines/chemokines**	**Secretion capacity of sWAT depot**		
	Chow	HFD+sham	HFD+WATx
**Adiponectin *(ng)***	**965.2 ± 498.0[a]**	**3154.3 ± 645.7[b]**	**3076.8 ± 1331.3[a,b]**
**Resistin *(ng)***	**112.2 ± 45.6[a]**	**247.1 ± 89.5[b]**	**249.6 ± 159.0[a,b]**
KC/CXCL1 *(pg)*	37116.9 ± 29755.8	122337.2 ± 83508.0	111767.5 ± 55573.0
**Leptin *(ng)***	**47.9 ± 45.3[a]**	**240.8 ± 161.6[b]**	**266.9 ± 145.4[b]**
MCP-1/CCL2 (*ng*)	6.7 ± 6.3	16.3 ± 13.5	13.9 ± 8.1
**IP-10/CXCL10 *(pg)***	**1695.3 ± 3377.0[a]**	**5602.7 ± 2735.3[b]**	**3083.3 ± 2768.6[a,b]**

**PAI-1 (*pg*)**	**2908.7 ± 3145.1[a]**	**8313.0 ± 4779.9[b]**	**7393.5 ± 4246.1[a,b]**
IL-10 *(pg)*	86.0 ± 62.5	670.4 ± 915.8	518.6 ± 346.9
MIP-1α/CCL3 *(pg)*	68.3 ± 80.5	1333.2 ± 2484.5	117.1 ± 58.0
TNF-α *(pg)*	58.2 ± 26.7	193.2 ± 178.7	326.0 ± 275.3
RANTES/CCL5 *(pg)*	159.1 ± 149.8	445.5 ± 315.9	523.5 ± 299.9
IL-17 *(pg)*	18.5 ± 12	87.6 ± 47.7	109.5 ± 111.7

Concentrations of adipokines and inflammatory factors measured in culture media after 4 hours of ex-vivo culture of the sWAT depots collected at the end of the study (t=28 weeks). Data are shown as mean ± SD. Statistical differences are indicated in bold; groups with the same superscript letters are statistically comparable (p>0.05) and groups with different superscript letters are statistically different (p≤0.05). Abbreviations: (IL-10) interleukin 10; (IL-17) interleukin 17; (IP-10/CXCL10) interferon ɣ-inducible protein 10; (KC/CXCL1) keratinocyte chemoattractant; (MCP-1/CCL2) monocyte chemoattractant protein 1; (MIP-1α/CCL3) macrophage inflammatory protein 1α; (PAI-1) plasminogen activator inhibitor 1; (RANTES/CCL5) regulated on activation, normal T-cell expressed and secreted; (TNF-α) tumor necrosis factor α.

S4 Table: Plasma protein levels measured by ELISA at t=27 weeks.

**Marker**	**Plasma**		
	Chow	HFD+sham	HFD+WATx
**Leptin *(ng/ml)***	**12.0 ± 6.7[a]**	**45.7 ± 9.3[b]**	**45.9 ± 12.9[b]**
Adiponectin *(μg/ml)*	15.0 ± 5.2	14.4 ± 7.9	11.5 ± 2.2
Resistin *(ng/ml)*	15.2 ± 6.1	14.8 ± 3.1	14.6 ± 3.4
**SAA *(μg/ml)***	**84.0 ± 180.8[a]**	**84.4 ± 60.8[b]**	**80.5 ± 70.1[b]**
**S100B *(pg/ml)***	**22.9 ± 10.1[a]**	**65.5 ± 24.2[b]**	**70.8 ± 28.7[b]**
**MIF *(ng/ml)***	**10.5 ± 7.4[a]**	**62.7 ± 19.1[b]**	**64.6 ± 14.7[b]**
**PAI-1 *(pg/ml)***	**269.4 ± 432.8[a]**	**500.5 ± 202.8[b]**	**539.5 ± 436.9[b]**
**IFN-ɣ *(pg/ml)***	**0.5 ± 0.2[a]**	**0.3 ± 0.1[b]**	**0.3 ± 0.2[b]**
**IL-10 *(pg/ml)***	**11.4 ± 3[a]**	**25.1 ± 12.2[b]**	**23.2 ± 8.4[b]**
IL-1β *(pg/ml)*	0.7 ± 0.7	0.6 ± 0.3	0.5 ± 0.2
IL-2 *(pg/ml)*	1.4 ± 0.4	1.4 ± 0.3	1.2 ± 0.3
IL-6 *(pg/ml)*	29.6 ± 41.1	28.0 ± 15.7	28.8 ± 13.7
**KC/CXCL1 *(pg/ml)***	**88.4 ± 47.8[a]**	**168.1 ± 79.2[b]**	**188.9 ± 83.0[b]**
**TNF-α *(pg/ml)***	**7.0 ± 2.7[a]**	**18.6 ± 7[b]**	**15 ± 6.5[b]**
IL-17A/F *(pg/ml)*	0.2 ± 0.2	0.4 ± 0.4	0.3 ± 0.2
IL-27-p28 *(pg/ml)*	2.4 ± 1.8	5.6 ± 5.0	6.9 ± 8.5
IL-33 *(pg/ml)*	2.4 ± 2.9	2.6 ± 3.2	3.7 ± 7.9
**IP-10/CXCL10 *(pg/ml)***	**35.3 ± 9.2[a]**	**61.5 ± 19.7[b]**	**54.6 ± 16.1[b]**
**MCP-1/CCL2 *(pg/ml)***	**15.7 ± 6.1[a]**	**41.3 ± 15.3[b]**	**37.6 ± 17.5[b]**

Plasma markers were measured at t=28 weeks (4 months after surgery). Data are shown as mean ± SD. Statistical differences are indicated in bold; groups with the same superscript letters are statistically comparable (p>0.05) and groups with different superscript letters are statistically different (p≤0.05). Abbreviations: (IFN-ɣ) interferon ɣ; (IL) interleukin; ; (IP-10/CXCL10) interferon ɣ-inducible protein 10; (KC/CXCL1) keratinocyte chemoattractant; (MCP-1/CCL2) monocyte chemoattractant protein 1; (MIF) macrophage migration inhibitory factor; (PAI-1) plasminogen activator inhibitor 1; (SAA) serum amyloid A; (TNF-α) tumor necrosis factor α.

S5 Table: Plasma protein levels measured by multiplex analysis at t=27 weeks.

Protein	**Concentrations (pg/ml)**		
	Chow	HFD+sham	HFD+WATx
Eotaxin-1/CCL11	827.07 ± 255.18	1102.47 ± 270.57	1010.89 ± 242.12
MCP-5/CCL12	191.12 ± 49.31	290.88 ± 69.75	303.02 ± 107.21
TARC/CCL17	83.38 ± 51.66	114.66 ± 54.74	77.16 ± 41.39
MCP-1/CCL2	403.7 ± 198.86	1172.15 ± 522.49	1081.14 ± 446.13
MDC/CCL22	132.05 ± 45.57	158.96 ± 58.52	143.84 ± 52.18
MIP-1β/CCL4	25.6 ± 5.76	58.23 ± 14.55	66.5 ± 25.46
RANTES/CCL5	31.86 ± 11.92	43.19 ± 20.09	33.73 ± 9.1
PD-L1/CD274	61.22 ± 17.9	86.98 ± 19.44	81.91 ± 13.46
M-CSF/CSF1	3842.35 ± 799.95	4150.31 ± 921.74	4360.93 ± 670.25
GM-CSF/CSF2	0.1 ± 0.03	0.13 ± 0.06	0.12 ± 0.05
G-CSF/CSF3	54.77 ± 22.66	60.47 ± 23.83	73.56 ± 30.02
CTLA-4	0.47 ± 0.1	0.58 ± 0.17	0.53 ± 0.08
KC/CXCL1	78.03 ± 21.39	155.79 ± 46.69	165.96 ± 65.52
I-TAC/CXCL11	0.03 ± 0.02	0.05 ± 0.03	0.06 ± 0.02
MIP-2/CXCL2	4.63 ± 2.15	13.31 ± 6.58	12.84 ± 5.82
MIG/CXCL9	154.97 ± 65.71	342.41 ± 126.33	306.63 ± 133.65
FGF21	500.78 ± 535.22	1722.91 ± 1181.5	1585.8 ± 1236.76
HGF	640.34 ± 493.59	860.29 ± 1114.62	474.66 ± 476.4
IFN-α2	0.12 ± 0.03	0.22 ± 0.08	0.27 ± 0.06
IFN-ɣ	0.45 ± 0.16	0.32 ± 0.09	0.27 ± 0.1
IFN- λ2	0.23 ± 0.06	0.38 ± 0.31	0.37 ± 0.15
IL-10	3.96 ± 1.02	8.77 ± 2.23	9.77 ± 3.38
IL-12α/IL-12β	1.38 ± 1.39	0.3 ± 0.26	0.41 ± 0.36
IL-16	2221.23 ± 405.21	2646.52 ± 706.27	2720.94 ± 863.72
IL-17A	1.47 ± 1.21	1.66 ± 0.85	2.22 ± 1.2
IL-17F	13.59 ± 8.9	32.23 ± 26.73	39.61 ± 30.76
IL-1α	130.76 ± 110.75	440.4 ± 396.38	384.01 ± 323.23
IL-1β	0.34 ± 0.22	0.49 ± 0.34	0.43 ± 0.23
IL-2	1.32 ± 0.31	1.62 ± 0.49	1.48 ± 0.39
IL-21	1.28 ± 1.11	0.86 ± 0.51	1.86 ± 1.05
IL-22	16.35 ± 8.64	10.96 ± 11.36	6.6 ± 2.79
IL-27	3.49 ± 1.64	8.3 ± 3.05	8.29 ± 3.66
IL-3	0.04 ± 0.04	0.06 ± 0.08	0.08 ± 0.03
IL-33	0.99 ± 1.09	1.6 ± 0.9	1.05 ± 0.86
IL-4	0.14 ± 0.04	0.19 ± 0.12	0.18 ± 0.07
IL-5	0.28 ± 0.12	0.47 ± 0.47	0.31 ± 0.07
IL-6	11.87 ± 17.73	39.21 ± 51.36	40.82 ± 28.5
IL-7	0.74 ± 0.39	0.93 ± 0.56	1.05 ± 0.48
IL-9	1.75 ± 1.38	4.38 ± 8.57	1.11 ± 0.4
PD-L2/PDCD1LG2	2264.68 ± 595.27	2364.98 ± 476.05	2396.86 ± 436.04
TNF	6.39 ± 3.29	13.75 ± 4.91	13.83 ± 4.93

Multiplexed quantification of proteins was performed in plasma samples collected t=27 weeks. Protein levels are expressed in Normalized Protein expression (NPX) units on a log2 scale. Data are shown as mean ± SD. Abbreviations: (CTLA-4) cytotoxic T-lymphocyte associated protein 4, (FGF21) fibroblast growth factor 2, (G-CSF/CSF3) granulocyte colony-stimulating factor, (GM-CSF/CSF2) granulocyte-macrophage colony-stimulating factor, (HGF) hepatocyte growth factor, (I-TAC/CXCL11) interferon-inducible T-cell alpha chemoattractant, (IFN) interferon, (IL) interleukin, (KC/CXCL1) keratinocyte-derived chemokine, (M-CSF/CSF1) macrophage colony-stimulating factor, (MCP-1/CCL2) monocyte chemoattractant protein 1, (MCP-5/CCL12) monocyte chemoattractant protein 5, (MDC/CCL22) macrophage-derived chemokine, (MIG/CXCL9) monokine induced by gamma interferon, (MIP-1β/CCL4) macrophage inflammatory protein 1β, (MIP-2/CXCL2) macrophage inflammatory protein 2,

(PD-L1/CD274) programmed death-ligand 1, (PD-L2/PDCD1LG2) programmed death-ligand 2, (RANTES/CCL5) regulated on activation, normal T-cell expressed and secreted, (TARC/CCL17) thymus and activation-regulated chemokine, (TNF) tumor necrosis factor.

S6 Table: Protein concentrations measured in brain cortex homogenates.

Marker (pg/mg tissue)	Brain cortex		
	Chow	HFD+sham	HFD+WATx
BDNF	1.93 ± 1.41	1.54 ± 0.57	1.27 ± 0.2
IL-10	0.12 ± 0.09	0.1 ± 0.07	0.08 ± 0.03
IL-15	2.97 ± 2.28	2.22 ± 1.16	1.68 ± 0.51
IL-1β	0.11 ± 0.07	0.10 ± 0.06	0.08 ± 0.03
IL-33	37.78 ± 15.40	46.13 ± 10.86	42.21 ± 9.69
IL-6	0.22 ± 0.17	0.17 ± 0.12	0.14 ± 0.04
MIP-1α/CCL3	0.54 ± 0.08	0.64 ± 0.15	0.55 ± 0.12
TNF-α	0.02 ± 0.02	0.02 ± 0.01	0.01 ± 0.00

Protein concentrations were measured by multiplex analysis in brain cortex homogenates at t=28 weeks (4 months after surgery). Data are shown as mean ± SD. Abbreviations: (BDNF) brain-derived neurotrophic factor, (IL) interleukin, (MIP-1α/CCL3) macrophage inflammatory protein-1α, (TNF-α) tumor necrosis factor α.

S7 Table: Canonical pathways analysis based on hippocampal gene expression in HFD+sham vs Chow assessed at t=28 weeks (4 months after surgery).

Pathway	-log(P)	z-score
Complement System	8.5	1.3
Role of Pattern Recognition Receptors in Recognition of Bacteria and Viruses	6.2	2.4
Fcγ Receptor-mediated Phagocytosis in Macrophages and Monocytes	5.7	2.6
Phagosome Formation	5.1	3.0
Neutrophil Extracellular Trap Signaling Pathway	4.9	1.2
Production of Nitric Oxide and Reactive Oxygen Species in Macrophages	4.5	1.6
Acute Phase Response Signaling	3.8	N/A
Leukocyte Extravasation Signaling	3.6	1.9
HER-2 Signaling in Breast Cancer	3.2	2.4
Th1 and Th2 Activation Pathway	3.1	N/A
Fc Epsilon RI Signaling	3.0	1.3
Actin Cytoskeleton Signaling	3.0	1.3
Neuroinflammation Signaling Pathway	3.0	2.4
LXR/RXR Activation	3.0	-1.3
Macrophage Classical Activation Signaling Pathway	2.9	2.4
Natural Killer Cell Signaling	2.8	0.8
ID1 Signaling Pathway	2.8	1.6
Th2 Pathway	2.8	0.0
Pyroptosis Signaling Pathway	2.5	2.0
Multiple Sclerosis Signaling Pathway	2.5	2.4

Phagosome Maturation	2.5	N/A
Breast Cancer Regulation by Stathmin1	2.4	1.9
Telomerase Signaling	2.3	N/A
IL-13 Signaling Pathway	2.2	2.0
Signaling by Rho Family GTPases	2.2	2.0
Th1 Pathway	2.1	N/A
GP6 Signaling Pathway	2.1	N/A
Pathogen Induced Cytokine Storm Signaling Pathway	2.0	2.6
fMLP Signaling in Neutrophils	2.0	2.0
GM-CSF Signaling	2.0	N/A

Only significantly enriched canonical pathways are displayed (p ≤ 0.01 (-log(P-value) ≥ 2)). The Z-score indicates the predicted activation of a canonical pathway: Z-score ≤ -2 indicates relevant inhibition of the pathway (shown in dark blue); Z-score ≥ 2 indicates relevant activation of the pathway (shown in dark red).

S8 Table: Upstream regulator analysis based on hippocampal gene expression in HFD+sham vs Chow assessed at t=28 weeks (4 months after surgery).

Upstream regulator	-log(P)	z-score
SPI1	14.1	3.5
GRN	12.3	-2.0
TCL1A	10.9	N/A
Immunoglobulin	10.9	-0.8
MAPT	10.7	N/A
KDM1A	10.4	N/A
HNRNPU	9.6	-3.0
USP22	9.2	1.0
PNPT1	9.0	-2.8
IFNG	8.9	4.1
B4GALNT1	7.9	-2.2
ITPR2	7.6	2.8
G protein alpha i	7.5	2.8
ST8SIA1	6.7	-2.2
IL10	6.4	2.0
JAK1	6.3	0.8
TNF	6.2	3.3
CSF1	6.2	2.0
TRIM24	6.1	-2.8
TREX1	6.1	-2.6
STAT1	6.1	3.2
RNASEH2B	6.1	-2.8
Interferon alpha	5.9	3.2
CNTF	5.8	2.3
SLC15A4	5.6	2.8

Ifnar	5.4	2.6
miR-219a-5p (and other miRNAs w/seed GAUUGUC)	5.3	N/A
STAG2	5.1	-2.2
CCL20	5.1	2.2
PCGF6	5.1	0.1
DUSP11	5.0	-2.0
BHLHE40	5.0	3.3
CSF2	5.0	2.3
CYP2E1	4.9	2.0
KRAS	4.8	N/A
IRF7	4.7	2.8
NR1H3	4.6	N/A
DRD2	4.6	1.7
LDLR	4.5	N/A
IFNA2	4.5	2.6
SOCS1	4.4	-2.4
AGT	4.4	3.8
GSDMB	4.4	N/A
Irgm1	4.4	-2.4
DNASE2	4.3	N/A
TYROBP	4.3	N/A
IL4	4.3	-0.5
Ttc39aos1	4.2	-2.2
PTGER4	4.1	-2.0
SPRY2	4.1	-1.3
APP	4.1	1.8
NCSTN	4.1	-2.0
PTPN11	4.1	-1.4
PTPN6	4.0	-0.3
IL6	4.0	0.7
RNASEL	4.0	N/A
ELOVL3	4.0	2.2
STAT3	4.0	0.4
KLK1	3.9	N/A
ETV5	3.9	-0.6
TGM2	3.8	2.8
IL21	3.8	1.6
FZD9	3.8	N/A
NKX2-1	3.7	N/A
SENP3	3.7	2.0
Hbb-b2	3.7	2.0
PSEN1	3.6	-2.2
ADIPOQ	3.6	-1.3
HRG	3.6	N/A

IRF8	3.6	-0.1
ACKR2	3.6	-2.0
RNASEH2A	3.5	N/A
Ige	3.5	3.0
Pka	3.5	2.2
Hbb-b1	3.5	2.2
SNCA	3.5	1.3
MYD88	3.4	1.8
Tcf7	3.4	-1.5
KLF6	3.4	2.2
IRF3	3.4	2.4
IL33	3.3	1.0
CX3CL1	3.3	2.2
EIF4E	3.3	1.9
GPR174	3.3	-0.4
TNFSF11	3.3	1.9
NRAS	3.3	-2.0
TLR7	3.3	2.4
ZBTB10	3.2	2.4
CLEC12A	3.2	N/A
CSF3	3.2	2.2
STAT2	3.2	N/A
SIRT1	3.2	-3.1
CITED2	3.2	-2.6
ETS1	3.2	2.2
CEBPB	3.2	-1.0
TBK1	3.1	N/A
APOE	3.1	-2.8
PPARD	3.1	0.4
ELF1	3.1	N/A
TRIM14	3.1	N/A
CCR2	3.1	2.2
KIT	3.0	N/A
IRAK3	3.0	N/A
DIO3	3.0	-0.8
IL13	3.0	0.1
IL10RA	3.0	-0.7
AIRE	3.0	N/A
TLR9	3.0	1.0
ABCC1	2.9	N/A
TGFBR1	2.9	-2.2
USP8	2.9	-2.0
JAK1/2	2.9	2.0
PRL	2.9	1.5

C1QA	2.9	N/A
TMEM120A	2.8	N/A
LY86	2.8	N/A
SMARCA5	2.8	-2.2
PLCG2	2.8	-1.0
AIM2	2.8	N/A
Ap2	2.8	N/A
NR5A2	2.8	0.8
NONO	2.8	2.2
PRDM16	2.7	N/A
Tnf (family)	2.7	-1.1
MEF2A	2.7	2.0
IRF2	2.7	-0.2
RUNX1	2.7	N/A
JUND	2.7	N/A
mir-21	2.7	-0.7
EEF1A2	2.7	N/A
C3	2.7	0.7
STING1	2.6	2.2
mir-155	2.6	-2.2
IRF1	2.6	1.4
IFNL1	2.6	2.0
CGAS	2.6	N/A
RARA	2.6	-1.0
CASP4	2.6	N/A
TP53	2.6	0.3
LMO2	2.6	-1.9
MIF	2.5	-2.2
ILF3	2.5	2.0
FLT3	2.5	N/A
DUSP1	2.5	-2.2
LDB1	2.5	-1.9
PRDM1	2.5	-1.1
IFNAR2	2.5	N/A
JAK2	2.5	N/A
IFNB1	2.5	2.2
EHF	2.5	-0.3
DYSF	2.5	N/A
ADAR	2.4	N/A
Hsp27	2.4	N/A
OLR1	2.4	N/A
STAT6	2.4	-0.1
PPARA	2.4	-0.4
HOXA3	2.4	N/A

IFNAR1	2.4	N/A
NCOR1	2.4	N/A
UCP1	2.4	-2.2
RELA	2.4	2.2
PSEN2	2.4	-2.0
IFIH1	2.3	N/A
CSK	2.3	N/A
FCGR1A	2.3	N/A
miR-125b-5p (and other miRNAs w/seed CCCUGAG)	2.3	-2.0
CYP19A1	2.3	N/A
BAX	2.3	N/A
ANXA1	2.3	N/A
TNK1	2.3	N/A
FGF10	2.3	N/A
CEBPE	2.3	N/A
FCGR2A	2.3	N/A
HIVEP1	2.3	N/A
IFN Beta	2.3	2.4
TP73	2.3	-0.3
IRF9	2.2	N/A
INSIG1	2.2	N/A
RARB	2.2	-0.7
SOD3	2.2	N/A
HRAS	2.2	0.7
CLEC11A	2.2	N/A
RGS10	2.2	N/A
TGFB1	2.2	2.7
PTPRM	2.2	N/A
4930481B07Rik	2.2	N/A
ARR3	2.2	N/A
Ctla2a/Ctla2b	2.2	N/A
FBXO46	2.2	N/A
GPLD1	2.2	N/A
RP11_750H95	2.2	N/A
TSKU	2.2	N/A
ZNF433	2.2	N/A
CLEC4A	2.2	N/A
CSF3R	2.2	N/A
FOXO4	2.2	-2.0
GIP	2.2	N/A
UBD	2.1	N/A
BID	2.1	N/A
MAFB	2.1	1.0
OSMR	2.1	N/A

MVP	2.1	N/A
MSC	2.1	N/A
PSMB11	2.1	-2.0
IL1B	2.1	2.0
GSTO1	2.1	N/A
SFTPD	2.1	N/A
Pde4	2.0	N/A
Cyp2c70	2.0	N/A
FLI1	2.0	N/A

Only significantly enriched upstream regulators are displayed (p ≤ 0.01 (-log(P-value) ≥ 2)). The Z-score indicates the predicted regulation of an upstream regulator: Z-score ≤ -2 indicates relevant downregulation of the upstream regulator (shown in dark blue); Z-score ≥ 2 indicates relevant upregulation of the upstream regulator (shown in dark red).

S9 Table: Upstream regulator analysis based on hippocampal gene expression in HFD+WATx vs HFD+sham assessed at t=28 weeks (4 months after surgery).

Upstream regulator	-log(P)	z-score
USP22	3.6	-2.0
miR-139-5p (miRNAs w/seed CUACAGU)	3.4	N/A
CKAP2L	3.2	N/A
BNIP3L	3.0	N/A
GATA2	2.5	N/A
AREG	2.5	N/A
PARP2	2.5	N/A
ATM/ATR	2.5	N/A
miR-99a-3p (and other miRNAs w/seed AAGCUCG)	2.5	N/A
MIR4500	2.5	N/A
MIR5095	2.5	N/A
TBRG4	2.5	N/A
Cbr2	2.5	N/A
mir-450	2.5	N/A
miR-450b-5p (miRNAs w/seed UUUGCAG)	2.5	N/A
miR-502-5p (and other miRNAs w/seed UCCUUGC)	2.5	N/A
PTGR2	2.5	N/A
ANXA13	2.5	N/A
IL2	2.4	N/A
DNASE2	2.3	N/A
TLR7	2.2	N/A
IFIT1B	2.2	N/A
RETSAT	2.2	N/A
Hoxaas3	2.2	N/A
KLHL40	2.2	N/A
KMT5A	2.2	N/A
L3MBTL1	2.2	N/A

mir-202	2.2	N/A
NEUROG2	2.2	N/A
E2F4	2.2	N/A
E2F1	2.1	N/A
FLT3	2.0	N/A
RYBP	2.0	N/A
FXR1	2.0	N/A

Only significantly enriched upstream regulators are displayed ($p \leq 0.01$ (-log(P-value) ≥ 2)). The Z-score indicates the predicted regulation of an upstream regulator: Z-score ≤ -2 indicates relevant downregulation of the upstream regulator (shown in dark blue).
