# Supplementary material for: Partial removal of visceral epididymal white adipose tissue in obese Ldlr-/-.Leiden mice impacts adipokine secretion, plasma free fatty acids, and improves cerebrovascular health
Source: PLoS One. 2025 Oct 17;20(10):e0333024. doi: 10.1371/journal.pone.0333024 (PMC12533877; doi:10.1371/journal.pone.0333024)
Supplement: Attachment — Submitted filename: Response_to_reviewers_PONE-D-25-13042.docx [file pone.0333024.s005.docx]

**PONE-D-25-13042**

**New title: Partial removal of visceral epididymal white adipose tissue in obese Ldlr-/-.Leiden mice impacts adipokine secretion, plasma free fatty acids, and improves cerebrovascular health**

**Response to editor and reviewer**

We would like to thank the editor and reviewers for the thoughtful comments and suggestions to improve our manuscript. We have provided an answer for each point below. The comments from the editor / reviewers are in black, followed by our answer and the corresponding changes as added in the manuscript in blue. In the revised manuscript itself, these edits appear as “tracked changes”.

Journal Requirements:

When submitting your revision, we need you to address these additional requirements.

1. Please ensure that your manuscript meets PLOS ONE's style requirements, including those for file naming. The PLOS ONE style templates can be found at <https://journals.plos.org/plosone/s/file?id=wjVg/PLOSOne_formatting_sample_main_body.pdf> and <https://journals.plos.org/plosone/s/file?id=ba62/PLOSOne_formatting_sample_title_authors_affiliations.pdf>

**Response from the authors:** We adjusted the manuscript to PLOS ONE’s style requirements as requested.

2. We note that you have indicated that there are restrictions to data sharing for this study. PLOS only allows data to be available upon request if there are legal or ethical restrictions on sharing data publicly. For more information on unacceptable data access restrictions, please see <http://journals.plos.org/plosone/s/data-availability#loc-unacceptable-data-access-restrictions>.

Before we proceed with your manuscript, please address the following prompts:

a) If there are ethical or legal restrictions on sharing a de-identified data set, please explain them in detail (e.g., data contain potentially identifying or sensitive patient information, data are owned by a third-party organization, etc.) and who has imposed them (e.g., a Research Ethics Committee or Institutional Review Board, etc.). Please also provide contact information for a data access committee, ethics committee, or other institutional body to which data requests may be sent.

b) If there are no restrictions, please upload the minimal anonymized data set necessary to replicate your study findings to a stable, public repository and provide us with the relevant URLs, DOIs, or accession numbers. For a list of recommended repositories, please see

<https://journals.plos.org/plosone/s/recommended-repositories>. You also have the option of uploading the data as Supporting Information files, but we would recommend depositing data directly to a data repository if possible.

We will update your Data Availability statement on your behalf to reflect the information you provide.

**Response from the authors:** The datasets are now publicly available in figshare repository (DOI: . <https://doi.org/10.6084/m9.figshare.29520716.v4>)

3. In the online submission form, you indicated that Datasets are available upon request and hippocampal RNAseq data will be available in the Gene Expression Omnibus (GEO) repository (https://www.ncbi.nlm.nih.gov/gds) after acceptance.

All PLOS journals now require all data underlying the findings described in their manuscript to be freely available to other researchers, either 1. In a public repository, 2. Within the manuscript itself, or 3. Uploaded as supplementary information.

This policy applies to all data except where public deposition would breach compliance with the protocol approved by your research ethics board. If your data cannot be made publicly available for ethical or legal reasons (e.g., public availability would compromise patient privacy), please explain your reasons on resubmission and your exemption request will be escalated for approval.

**Response from the authors:** The RNAseq dataset is now publicly available in the NCBI database under the accession number GSE300930. We corrected the corresponding statements in the revised manuscript and provided a link for readers to access the dataset.

Text in the Methods (lines 306-308 in the revised manuscript with tracked changes): “The transcriptomics dataset generated for this study is publicly available in the Gene Expression Omnibus (GEO) repository (#GSE300930, <https://www.ncbi.nlm.nih.gov/gds>).”

4. Please amend your list of authors on the manuscript to ensure that each author is linked to an affiliation. Authors’ affiliations should reflect the institution where the work was done (if authors moved subsequently, you can also list the new affiliation stating “current affiliation:….” as necessary).

**Response from the authors:** We amended the list of authors and the list is now correct. Please note that in the course of the revision three new authors were included in the study (E.G., S.E., J.B.) due to their contribution regarding the plasma free fatty acid measurements and the multiplex Olink experiments. These authors are now listed in the revised author line. All authors agree with the revised author list.

5. We note that you have included the phrase “data not shown” in your manuscript. Unfortunately, this does not meet our data sharing requirements. PLOS does not permit references to inaccessible data. We require that authors provide all relevant data within the paper, Supporting Information files, or in an acceptable, public repository. Please add a citation to support this phrase or upload the data that corresponds with these findings to a stable repository (such as Figshare or Dryad) and provide and URLs, DOIs, or accession numbers that may be used to access these data. Or, if the data are not a core part of the research being presented in your study, we ask that you remove the phrase that refers to these data.

**Response from the authors:** We added the corresponding data in the Supplementary material (Supplementary S2 Table and S3 Table).

New S2 Table:

**S2 Table: Inflammatory proteins secreted within 4 hours by mWAT depots at the end of the study.**

| **Cytokines/chemokines** |  | | **Secretion capacity of mWAT depot** | | |
| --- | --- | --- | --- | --- | --- |
|  |  | | Chow | HFD+sham | HFD+WATx |
| **Adiponectin *(ng)*** |  | | **1065.8 ± 389.5^a^** | **1863.9 ± 252.0^b^** | **1630.6 ± 455.0^b^** |
| Resistin *(ng)* |  | | 148.2 ± 59.1 | 101.1 ± 58.1 | 99.7 ± 48.4 |
| KC/CXCL1 *(pg)* |  | 155021.2 ± 66776.2 | | 200146.2 ± 165678.4 | 306516.8 ± 233316 |
| **Leptin *(ng)*** |  | | **24.9 ± 29.2^a^** | **73.1 ± 39.9^b^** | **84.2 ± 54.2^b^** |
| MCP-1/CCL2 (*ng*) |  | | 15.6 ± 6.3 | 19.9 ± 16.7 | 17.5 ± 17.2 |
| IP-10/CXCL10 *(pg)* |  | | 10085.6 ± 6843.7 | 6016.8 ± 4988.8 | 8761.8 ± 4353.8 |
| PAI-1 (*pg*) |  | | 6822.0 ± 5025.1 | 6167.0 ± 4306.5 | 4878.9 ± 1546.6 |
| IL-10 *(pg)* |  | | 922.7 ± 540.5 | 850.4 ± 803.8 | 2098.4 ± 2158.4 |
| MIP-1α/CCL3 *(pg)* |  | | 285.8 ± 183.2 | 471.3 ± 682.4 | 433.9 ± 475.2 |
| TNF-α *(pg)* |  | | 226.6 ± 113.5 | 152.3 ± 118.3 | 473.5 ± 470.2 |
| RANTES/CCL5 *(pg)* |  | | 275.9 ± 191.6 | 268.5 ± 130.5 | 497.1 ± 228.0 |
| IL-17 *(pg)* |  | | n.d. | n.d. | n.d. |

Concentrations of adipokines and inflammatory factors measured in culture media after 4 hours of ex-vivo culture of the mWAT depots collected at the end of the study (t=28 weeks). IL-17 data were excluded as most values were below the detection level (n.d.). Data for all other factors are shown as mean ± SD. Statistical differences are indicated in bold; groups with the same superscript letters are statistically comparable (p>0.05) and groups with different superscript letters are statistically different (p≤0.05). Abbreviations: (IL-10) interleukin 10; (IL-17) interleukin 17; (IP-10/CXCL10) interferon ɣ-inducible protein 10; (KC/CXCL1) keratinocyte chemoattractant; (MCP-1/CCL2) monocyte chemoattractant protein 1; (MIP-1α/CCL3) macrophage inflammatory protein 1α; (PAI-1) plasminogen activator inhibitor 1; (RANTES/CCL5) regulated on activation, normal T-cell expressed and secreted; (TNF-α) tumor necrosis factor α.

New S3 Table:

**S3 Table: Inflammatory proteins secreted within 4 hours by sWAT depots at the end of the study.**

| **Cytokines/chemokines** |  | **Secretion capacity of sWAT depot** | | |
| --- | --- | --- | --- | --- |
|  |  | Chow | HFD+sham | HFD+WATx |
| **Adiponectin *(ng)*** |  | **965.2 ± 498.0^a^** | **3154.3 ± 645.7^b^** | **3076.8 ± 1331.3^a,b^** |
| **Resistin *(ng)*** |  | **112.2 ± 45.6^a^** | **247.1 ± 89.5^b^** | **249.6 ± 159.0^a,b^** |
| KC/CXCL1 *(pg)* |  | 37116.9 ± 29755.8 | 122337.2 ± 83508.0 | 111767.5 ± 55573.0 |
| **Leptin *(ng)*** |  | **47.9 ± 45.3^a^** | **240.8 ± 161.6^b^** | **266.9 ± 145.4^b^** |
| MCP-1/CCL2 (*ng*) |  | 6.7 ± 6.3 | 16.3 ± 13.5 | 13.9 ± 8.1 |
| **IP-10/CXCL10 *(pg)*** |  | **1695.3 ± 3377.0^a^** | **5602.7 ± 2735.3^b^** | **3083.3 ± 2768.6^a,b^** |
| **PAI-1 (*pg*)** |  | **2908.7 ± 3145.1^a^** | **8313.0 ± 4779.9^b^** | **7393.5 ± 4246.1^a,b^** |
| IL-10 *(pg)* |  | 86.0 ± 62.5 | 670.4 ± 915.8 | 518.6 ± 346.9 |
| MIP-1α/CCL3 *(pg)* |  | 68.3 ± 80.5 | 1333.2 ± 2484.5 | 117.1 ± 58.0 |
| TNF-α *(pg)* |  | 58.2 ± 26.7 | 193.2 ± 178.7 | 326.0 ± 275.3 |
| RANTES/CCL5 *(pg)* |  | 159.1 ± 149.8 | 445.5 ± 315.9 | 523.5 ± 299.9 |
| IL-17 *(pg)* |  | 18.5 ± 12 | 87.6 ± 47.7 | 109.5 ± 111.7 |

Concentrations of adipokines and inflammatory factors measured in culture media after 4 hours of ex-vivo culture of the sWAT depots collected at the end of the study (t=28 weeks). Data are shown as mean ± SD. Statistical differences are indicated in bold; groups with the same superscript letters are statistically comparable (p>0.05) and groups with different superscript letters are statistically different (p≤0.05). Abbreviations: (IL-10) interleukin 10; (IL-17) interleukin 17; (IP-10/CXCL10) interferon ɣ-inducible protein 10; (KC/CXCL1) keratinocyte chemoattractant; (MCP-1/CCL2) monocyte chemoattractant protein 1; (MIP-1α/CCL3) macrophage inflammatory protein 1α; (PAI-1) plasminogen activator inhibitor 1; (RANTES/CCL5) regulated on activation, normal T-cell expressed and secreted; (TNF-α) tumor necrosis factor α.

6. Please include captions for your Supporting Information files at the end of your manuscript, and update any in-text citations to match accordingly. Please see our Supporting Information guidelines for more information: <http://journals.plos.org/plosone/s/supporting-information>.

**Response from the authors:** We added the caption for the Supporting Information file at the end of the manuscript.

Text added (lines 876-896 of the revised manuscript with track changes):

“**Supporting information**

S1 Fig: Liver disease and atherosclerosis at the end of the study.

S2 Fig: Histological analyses of mesenteric (mWAT) and subcutaneous (sWAT) WAT at the end of the study.

S3 Fig: Grey and white matter integrity.

S43 Fig: Spatial learning and (short-term) memory performance before surgery.

S5 Fig: Histological markers of neuroinflammation, vascular integrity and neurogenesis at the end of the study.

S6 Fig: Activation of canonical pathways and upstream regulators based on hippocampal gene expression.

S1 Table: Sample size per experiment.

S2 Table: Inflammatory proteins secreted within 4 hours by mWAT depots at the end of the study.

S3 Table: Inflammatory proteins secreted within 4 hours by sWAT depots at the end of the study.

S4 Table: Plasma protein levels measured by ELISA at t=27 weeks.

S5 Table: Plasma protein levels measured by multiplex analysis at t=27 weeks.

S6 Table: Protein concentrations measured in brain cortex homogenates.

S7 Table: Canonical pathways analysis based on hippocampal gene expression in HFD+sham vs Chow assessed at t=28 weeks (4 months after surgery).

S8 Table: Upstream regulator analysis based on hippocampal gene expression in HFD+sham vs Chow assessed at t=28 weeks (4 months after surgery).

S9 Table: Upstream regulator analysis based on hippocampal gene expression in HFD+WATx vs HFD+sham assessed at t=28 weeks (4 months after surgery).”

**Additional Editor Comments:**

**Comment from the Editor:** The evidence supporting improved hippocampal vasoreactivity and cortico-hippocampal connectivity post-lipectomy is compelling, backed by robust MRI and behavioral data. However, the cognitive outcomes are mixed, with no clear improvement in learning tasks despite enhanced connectivity. This discrepancy suggests that other factors (e.g., aging, additional comorbidities) may modulate cognitive outcomes. The histological absence of neuroinflammation contrasts with RNAseq findings, highlighting the need for complementary techniques (e.g., multiplex cytokine assays) to resolve this inconsistency.

**Response from the authors:** We thank the editor for this valuable point. To support the observations made in brain histopathological analyses and hippocampal RNAseq, and to begin to deepen insight into the apparent link between brain histology and hippocampal transcriptomic profile, we measured a selected set of eight inflammatory markers in brain cortex homogenates. Hippocampi were already completely used for our extensive histological analysis and the RNAseq analysis. Therefore, cortex samples were used as an alternative since this brain region is also linked to the cognitive outcomes analyzed in the present study (i.e., spatial learning and memory). The obtained results are now displayed in the new S6 Table. Consistent with the results regarding histologically-evaluated neuroinflammation, there were no differences between the chow-fed and high fat diet (HFD)-fed mice. This discrepancy with the RNAseq findings may be due to the fact that pathway and upstream regulator analyses predict the activation of neuroinflammatory pathways based on the activation and inhibition of hundreds of inflammatory factors, while in histological analyses it is only possible to measure a small number of proteins. In this respect (but beyond the scope of the present study), it would be interesting to perform a full proteomics analyses, preferably using time-course experimental set-up. This would enable us to identify molecules that are differentially regulated by HFD feeding compared with chow feeding, including, for example, transcription factors, enzymes or their regulators that control the production of oxylipins (i.e., pro/anti-inflammatory lipids such as prostaglandins) as they seem to be differentially expressed in the brains of Ldlr-/-.Leiden mice base on a recent study in which we compared different energy-dense diets with chow diet.

Of note, an analysis of the changes over time during HFD treatment may also be very important based on in-house scouting experiment: We analyzed brain homogenates from an in-house study in Ldlr-/-.Leiden mice with comparable experimental settings (n=8-15 mice). Male Ldlr-/-.Leiden mice were fed a chow diet for 34 weeks, or they were stimulated with a HFD either for a short (20 weeks) or a long (34 weeks) period. All animals started the HFD treatment at an age similar to the present study. At 20 weeks or 34 weeks respectively, the brains were collected as described in the present study, and similar markers were measured in hippocampi homogenates (figure below, unpublished data from scouting experiment). At 20 weeks of HFD feeding, we observed the highest concentrations of cytokines and chemokines, albeit with considerable variation. Thus, mice fed a HFD during 20 weeks showed higher concentrations of multiple cytokines/chemokines compared with chow-fed mice, and these inflammatory mediators seem to decline over time reaching comparable levels to chow at 34 weeks of HFD feeding. As this scouting experiment did not include a group of mice fed a chow diet during 20 weeks, we cannot draw firm conclusions but the data nevertheless suggest that inflammatory mediators have a dynamic expression pattern, and these dynamics may differ between diets. On HFD, the concentrations of inflammatory proteins seem to follow a bell-shaped curve, which may explain why differences between chow-fed and HFD-fed mice are not (*anymor*e) visible at later time points, as observed in the present manuscript. To gain insight in this, an extensive time course experiment with analyses of chow-fed and HFD-fed groups would be necessary, and we plan to apply for resources to conduct such a study.

New S6 table in current manuscript:

**S6 Table: Protein concentrations measured in brain cortex homogenates.**

| **Marker (pg/mg tissue)** | **Brain cortex** | | |
| --- | --- | --- | --- |
|  | Chow | HFD+sham | HFD+WATx |
| BDNF | 1.93 ± 1.41 | 1.54 ± 0.57 | 1.27 ± 0.2 |
| IL-10 | 0.12 ± 0.09 | 0.1 ± 0.07 | 0.08 ± 0.03 |
| IL-15 | 2.97 ± 2.28 | 2.22 ± 1.16 | 1.68 ± 0.51 |
| IL-1β | 0.11 ± 0.07 | 0.10 ± 0.06 | 0.08 ± 0.03 |
| IL-33 | 37.78 ± 15.40 | 46.13 ± 10.86 | 42.21 ± 9.69 |
| IL-6 | 0.22 ± 0.17 | 0.17 ± 0.12 | 0.14 ± 0.04 |
| MIP-1α/CCL3 | 0.54 ± 0.08 | 0.64 ± 0.15 | 0.55 ± 0.12 |
| TNF-α | 0.02 ± 0.02 | 0.02 ± 0.01 | 0.01 ± 0.00 |

Protein concentrations were measured by multiplex analysis in brain cortex homogenates at t=28 weeks (4 months after surgery). Data are shown as mean ± SD. Abbreviations: (BDNF) brain-derived neurotrophic factor, (IL) interleukin, (MIP-1α/CCL3) macrophage inflammatory protein-1α, (TNF-α) tumor necrosis factor α.

Text in the Methods (lines 291-295 in the revised manuscript with tracked changes): “**Protein concentrations in brain cortex homogenates.** Brain cortex homogenates were prepared in lysis buffer as previously described [1,2]. Concentrations of brain-derived neurotrophic factor (BDNF), IL-10, IL-15, IL-1β, IL-33, IL-6, MIP-1α/CCL3 and TNF-α were measured by multiplex analysis using a U-PLEX Custom Metabolic Group 1 set (Mesoscale discoveries [MSD], Maryland, USA). Protein levels were expressed per mg tissue.”

Text in the Results (lines 610-612 in the revised manuscript with tracked changes): “In parallel, multiplex-based quantification of cytokines (IL-10, IL-15, IL-1β, IL-33, IL-6, TNF-α) and other markers (BDNF, MIP-1α/CCL3) in brain cortex homogenates showed no significant differences between the three groups (S6 Table).”

Editor/Reviewer-only data (from unpublished in-house scouting experiment) suggesting dynamic expression changes upon HFD feeding:

**Figure: Effect of HFD feeding on protein expression of cytokines and other markers using brain homogenates from Ldlr-/-.Leiden mice (in-house data).** Markers were measured in brain hippocampus homogenates in mice fed a chow diet during 34 weeks, and mice fed a HFD for either 20 weeks or 34 weeks (n=8-15 animals per group). Data are shown as mean ± SD. Abbreviations: (BDNF) brain-derived neurotrophic factor, (IL) interleukin, (MIP-1α) macrophage inflammatory protein-1α, (TNF-α) tumor necrosis factor α.

**Comment #1 from the Editor:** Discuss how eWAT findings might extrapolate to human visceral fat, addressing anatomical/functional differences and potential compensatory mechanisms in humans.

**Response from the authors:** We thank the reviewer for raising this point. Translational aspects of the eWAT depot to human visceral WAT were already described in the Discussion of the initial manuscript, and we have now extended this paragraph to emphasize the anatomical and functional differences.

Text in the Discussion (lines 834-852 in the revised manuscript with tracked changes): “An important limitation of this study is whether mouse eWAT accurately translates to abdominal visceral WAT in humans. Given that omental WAT is minimally present in mice [3], numerous similarities have been identified between mouse eWAT and human omental WAT, particularly in their roles in metabolic regulation, cholinergic regulation, and inflammation [4,5]. Both depots can become inflamed (e.g., they develop CLS formed by immune cells that surround one or more defect adipocytes) and dysfunctional (e.g., hypertrophic adipocytes that release inflammatory factors including cytokines and lipids) in the context of obesity, contributing to systemic insulin resistance and metabolic comorbidities [6,7]. However, it is important to mention that mouse eWAT and human omental WAT have different anatomical location, biological function and draining system [5]. Although both depots contribute to the development of metabolic comorbidities through systemic inflammation and circulating metabolites (e.g., release of fatty acids), omental WAT may also influence nearby metabolic organs (e.g., the liver) through additional short-distance mechanisms due to its close anatomical proximity within the abdomen [8]. Furthermore, the lipectomy performed in this study in mice (eWAT removal) should not be compared to a standard lipectomy surgery performed in humans (typically removing subcutaneous WAT and not visceral WAT). It is likely that, upon lipectomy, other compensatory mechanisms take place in humans in comparison with mice. Thus, while eWAT lipectomy in mice provides valuable proof-of-concept insights regarding the role of a WAT depot with high secretory capacity (e.g., release of cytokines, lipids) for brain health, it is difficult to translate this to the human situation, and it remains important to consider the aforementioned anatomical and functional differences when translating findings to human physiology.”

**Comment #2 from the Editor:** Perform additional cognitive tests (e.g., fear conditioning) to reconcile behavioral findings with imaging results. Consider longer post-surgery follow-ups to capture delayed cognitive effects.

**Response from the authors:** We acknowledge that performing different cognitive tests would provide additional information regarding cognitive functions. However, our study is focused on hippocampal-related cognitive function, which is considered to be impaired in the context of obesity, and we therefore used cognitive tasks to test memory and spatial learning. In addition, the absence of effects on cognition upon eWAT lipectomy is probably not due to the limited number of cognitive tests performed nor the duration of post-surgery monitoring, but rather due to the fact that the effect of eWAT lipectomy on cortico-hippocampal connectivity and hippocampal vasoreactivity was very mild. Despite correlations between brain blood flow outcomes and cognitive performance in this mouse model [9], the effect of eWAT lipectomy may simply not be large enough to trigger changes in cognitive function. This may be explained by the observation that the eWAT lipectomy only modestly impacted the concentrations of pro-inflammatory factors (proteins and lipids) in the circulation. Furthermore, the effect of obesity on the brain is multifactorial and underlain by multiple complex processes involving other WAT depots and other organs. For example, the levels of plasma LDL-cholesterol have been related to dementia [10], and other factors that are not affected by eWAT lipectomy, such as severe insulin resistance (e.g., we still observed high insulin levels) and vascular dysfunction (e.g., development of atherosclerosis was not changed), may also maintain cognitive dysfunction [11,12]. While eWAT lipectomy did not have an effect on memory performance and spatial learning, it did impact behavior: Based on activity monitoring in Digitally Ventilated Cages, HFD+sham mice showed a decrease in activity during the night (period of activity in healthy mice), while this sedentary behavior was not observed in Chow controls nor in HFD+WATx mice. The mechanisms linking visceral WAT dysfunction, locomotor activity and circadian rhythms are however not yet explored. We discussed these elements in the Discussion of the revised manuscript.

Text in the Discussion (lines 790-805 in the revised manuscript with tracked changes): “However, this is a subtle observation and no clear effect of the eWAT lipectomy was observed on cognitive performance, which may be due to the fact that the effect of eWAT lipectomy on cortico-hippocampal connectivity and hippocampal vasoreactivity was very mild. Despite correlations between cerebral blood flow outcomes and cognitive performance in this mouse model [9], the effect of eWAT lipectomy may not be large enough to trigger significant changes in cognitive function. Furthermore, obesity effect on brain function is complex and multifactorial and other detrimental processes that were not affected by eWAT lipectomy in this study, such as insulin resistance, dyslipidemia (e.g., high LDL plasma levels), vascular dysfunction (e.g., atherosclerosis development), may still cause detrimental effects and hence maintain obesity-induced cognitive deficits [10–12].

Despite the absence of substantial effects on cognitive performance, the eWAT lipectomy affected important behavioral aspects: After 25 weeks of HFD feeding, HFD+sham mice exhibited reduced home-cage night activity towards their day-time activity levels (the inactive period in mice), indicative of a more sedentary behavior. Interestingly, the partial eWAT lipectomy prevented the development of this HFD-induced sedentary behavior as eWAT-lipectomized mice remained active at night similarly to Chow reference mice. Future studies are however needed to unravel potential mechanisms linking visceral WAT dysfunction, locomotor activity and circadian rhythms.”

**Comment #3 from the Editor:** Measure arteriovenous differences in adipokines/cytokines or use kinetic labeling to better quantify WAT-derived systemic contributions.

**Response from the authors:** We appreciate the editor's thoughtful suggestion and included this consideration in the Discussion section of the revised manuscript. However, it is important to note that conducting arteriovenous analyses would require sedation, which could affect plasma cytokine concentrations. As an alternative, we performed a refined multiplex quantification of multiple mouse proteins in plasma samples collected at t=27 weeks (4 months after surgery). Normalized Protein expression (NPX) values and corresponding absolute concentrations are now respectively reported in Table 4 and S5 Table. We observed elevated levels of several pro-inflammatory factors (e.g., CCL2 (MCP-1), CCL4 (MIP-1β), IL-1α, IL-3, IL-6, TNF), metabolic factors (e.g., fibroblast growth factor 2 (FGF21) and some anti-inflammatory factors (e.g., IL-10) upon HFD feeding. IL-2 levels were also specifically decreased with HFD feeding. Furthermore, the eWAT lipectomy triggered a reduction in IFN-ɣ levels and an increase in IL-17F levels when comparing HFD+WATx mice and Chow mice (but there were no significant differences in protein concentrations between HFD+WATx and HFD+sham mice).

New Table 4:

**Table 4: Multiplex analysis of plasma proteins at t=27 weeks (4 months after surgery).**

| Protein | **Normalized Protein Expression (NPX) units** | | |
| --- | --- | --- | --- |
|  | Chow | HFD+sham | HFD+WATx |
| Eotaxin-1/CCL11 | 10.78 ± 0.44 | 11.22 ± 0.35 | 11.09 ± 0.36 |
| **MCP-5/CCL12** | **7.98 ± 0.38^a^** | **8.65 ± 0.36^b^** | **8.67 ± 0.52^b^** |
| TARC/CCL17 | 2.76 ± 1.09 | 3.47 ± 0.98 | 2.67 ± 0.92 |
| **MCP-1/CCL2** | **10.76 ± 0.58^a^** | **12.16 ± 0.54^b^** | **12.05 ± 0.61^b^** |
| MDC/CCL22 | 12.33 ± 0.55 | 12.60 ± 0.53 | 12.45 ± 0.57 |
| **MIP-1β/CCL4** | **7.53 ± 0.31^a^** | **8.65 ± 0.37^b^** | **8.76 ± 0.57^b^** |
| RANTES/CCL5 | 5.63 ± 0.65 | 6.15 ± 0.75 | 5.79 ± 0.47 |
| **PD-L1/CD274** | **4.21 ± 0.34^a^** | **4.71 ± 0.31^b^** | **4.64 ± 0.24^b^** |
| M-CSF/CSF1 | 7.71 ± 0.33 | 7.82 ± 0.34 | 7.91 ± 0.22 |
| GM-CSF/CSF2 | 1.58 ± 0.24 | 1.75 ± 0.34 | 1.70 ± 0.43 |
| G-CSF/CSF3 | 7.30 ± 0.66 | 7.49 ± 0.51 | 7.78 ± 0.55 |
| CTLA-4 | 3.93 ± 0.29 | 4.19 ± 0.36 | 4.10 ± 0.21 |
| **KC/CXCL1** | **11.77 ± 0.36^a^** | **12.67 ± 0.39^b^** | **12.71 ± 0.5^b^** |
| I-TAC/CXCL11 | 0.44 ± 0.27 | 0.65 ± 0.3 | 0.77 ± 0.24 |
| **MIP-2/CXCL2** | **6.15 ± 0.58^a^** | **7.62 ± 0.58^b^** | **7.56 ± 0.71^b^** |
| **MIG/CXCL9** | **9.39 ± 0.51^a^** | **10.53 ± 0.51^b^** | **10.34 ± 0.61^b^** |
| **FGF21** | **10.58 ± 1.36^a^** | **12.70 ± 1.04^b^** | **12.45 ± 1.31^b^** |
| HGF | 9.41 ± 3.64 | 8.51 ± 4.69 | 7.46 ± 5.07 |
| **IFN-α2** | **3.14 ± 0.46^a^** | **3.9 ± 0.39^b^** | **4.18 ± 0.32^b^** |
| **IFN-ɣ** | **4.24 ± 0.51^a^** | **3.86 ± 0.34^a,b^** | **3.65 ± 0.35^b^** |
| **IFN- λ2** | **1.82 ± 0.23^a^** | **2.21 ± 0.58^b^** | **2.25 ± 0.41^b^** |
| **IL-10** | **3.38 ± 0.28^a^** | **4.39 ± 0.34^b^** | **4.49 ± 0.53^b^** |
| IL-12α/IL-12β | 2.66 ± 1.28 | 1.44 ± 0.64 | 1.62 ± 0.9 |
| IL-16 | 8.60 ± 0.27 | 8.84 ± 0.36 | 8.86 ± 0.43 |
| IL-17A | 5.30 ± 1.03 | 5.70 ± 0.61 | 6.05 ± 0.75 |
| **IL-17F** | **6.17 ± 1.35^a^** | **7.47 ± 1.18^a,b^** | **7.92 ± 0.94^b^** |
| **IL-1α** | **8.44 ± 1.13^a^** | **10.29 ± 1.08^b^** | **9.83 ± 1.59^b^** |
| IL-1β | 2.86 ± 0.59 | 3.23 ± 0.67 | 3.15 ± 0.55 |
| IL-2 | 2.93 ± 0.27 | 3.15 ± 0.34 | 3.06 ± 0.28 |
| IL-21 | 0.07 ± 0.38 | -0.02 ± 0.2 | 0.28 ± 0.27 |
| **IL-22** | **4.83 ± 0.72^a^** | **4.05 ± 0.98^b^** | **3.64 ± 0.49^b^** |
| **IL-27** | **2.35 ± 0.43^a^** | **3.36 ± 0.49^b^** | **3.35 ± 0.52^b^** |
| **IL-3** | **0.50 ± 0.48^a^** | **0.80 ± 0.46^a,b^** | **0.99 ± 0.26^b^** |
| IL-33 | 2.16 ± 0.87 | 2.83 ± 0.54 | 2.32 ± 0.75 |
| IL-4 | 1.21 ± 0.27 | 1.40 ± 0.41 | 1.39 ± 0.3 |
| IL-5 | 4.14 ± 0.55 | 4.62 ± 0.85 | 4.34 ± 0.33 |
| **IL-6** | **2.71 ± 1.12^a^** | **4.21 ± 1.1^b^** | **4.48 ± 0.96^b^** |
| IL-7 | 1.38 ± 0.45 | 1.52 ± 0.49 | 1.67 ± 0.36 |
| IL-9 | 4.83 ± 0.96 | 5.36 ± 1.47 | 4.43 ± 0.48 |
| PD-L2/PDCD1LG2 | 13.15 ± 0.30 | 13.21 ± 0.28 | 13.24 ± 0.23 |
| **TNF** | **3.45 ± 0.54^a^** | **4.65 ± 0.56^b^** | **4.66 ± 0.58^b^** |

Multiplexed quantification of proteins was performed in plasma samples collected t=27 weeks. Protein levels are expressed in Normalized Protein expression (NPX) units on a log2 scale. Data are shown as mean ± SD. Statistical differences are indicated in bold; groups with the same superscript letters are statistically comparable (p>0.05) and groups with different superscript letters are statistically different (p≤0.05). Abbreviations: (CTLA-4) cytotoxic T-lymphocyte associated protein 4, (FGF21) fibroblast growth factor 2, (G-CSF/CSF3) granulocyte colony-stimulating factor, (GM-CSF/CSF2) granulocyte-macrophage colony-stimulating factor, (HGF) hepatocyte growth factor, (I-TAC/CXCL11) interferon-inducible T-cell alpha chemoattractant, (IFN) interferon, (IL) interleukin, (KC/CXCL1) keratinocyte-derived chemokine, (M-CSF/CSF1) macrophage colony-stimulating factor, (MCP-1/CCL2) monocyte chemoattractant protein 1, (MCP-5/CCL12) monocyte chemoattractant protein 5, (MDC/CCL22) macrophage-derived chemokine, (MIG/CXCL9) monokine induced by gamma interferon, (MIP-1β/CCL4) macrophage inflammatory protein 1β, (MIP-2/CXCL2) macrophage inflammatory protein 2, (PD-L1/CD274) programmed death-ligand 1, (PD-L2/PDCD1LG2) programmed death-ligand 2, (RANTES/CCL5) regulated on activation, normal T-cell expressed and secreted, (TARC/CCL17) thymus and activation-regulated chemokine, (TNF) tumor necrosis factor.

New S5 table:

**S5 Table: Plasma protein levels measured by multiplex analysis at t=27 weeks.**

| Protein | **Concentrations (pg/ml)** | | |
| --- | --- | --- | --- |
|  | Chow | HFD+sham | HFD+WATx |
| Eotaxin-1/CCL11 | 827.07 ± 255.18 | 1102.47 ± 270.57 | 1010.89 ± 242.12 |
| MCP-5/CCL12 | 191.12 ± 49.31 | 290.88 ± 69.75 | 303.02 ± 107.21 |
| TARC/CCL17 | 83.38 ± 51.66 | 114.66 ± 54.74 | 77.16 ± 41.39 |
| MCP-1/CCL2 | 403.7 ± 198.86 | 1172.15 ± 522.49 | 1081.14 ± 446.13 |
| MDC/CCL22 | 132.05 ± 45.57 | 158.96 ± 58.52 | 143.84 ± 52.18 |
| MIP-1β/CCL4 | 25.6 ± 5.76 | 58.23 ± 14.55 | 66.5 ± 25.46 |
| RANTES/CCL5 | 31.86 ± 11.92 | 43.19 ± 20.09 | 33.73 ± 9.1 |
| PD-L1/CD274 | 61.22 ± 17.9 | 86.98 ± 19.44 | 81.91 ± 13.46 |
| M-CSF/CSF1 | 3842.35 ± 799.95 | 4150.31 ± 921.74 | 4360.93 ± 670.25 |
| GM-CSF/CSF2 | 0.1 ± 0.03 | 0.13 ± 0.06 | 0.12 ± 0.05 |
| G-CSF/CSF3 | 54.77 ± 22.66 | 60.47 ± 23.83 | 73.56 ± 30.02 |
| CTLA-4 | 0.47 ± 0.1 | 0.58 ± 0.17 | 0.53 ± 0.08 |
| KC/CXCL1 | 78.03 ± 21.39 | 155.79 ± 46.69 | 165.96 ± 65.52 |
| I-TAC/CXCL11 | 0.03 ± 0.02 | 0.05 ± 0.03 | 0.06 ± 0.02 |
| MIP-2/CXCL2 | 4.63 ± 2.15 | 13.31 ± 6.58 | 12.84 ± 5.82 |
| MIG/CXCL9 | 154.97 ± 65.71 | 342.41 ± 126.33 | 306.63 ± 133.65 |
| FGF21 | 500.78 ± 535.22 | 1722.91 ± 1181.5 | 1585.8 ± 1236.76 |
| HGF | 640.34 ± 493.59 | 860.29 ± 1114.62 | 474.66 ± 476.4 |
| IFN-α2 | 0.12 ± 0.03 | 0.22 ± 0.08 | 0.27 ± 0.06 |
| IFN-ɣ | 0.45 ± 0.16 | 0.32 ± 0.09 | 0.27 ± 0.1 |
| IFN- λ2 | 0.23 ± 0.06 | 0.38 ± 0.31 | 0.37 ± 0.15 |
| IL-10 | 3.96 ± 1.02 | 8.77 ± 2.23 | 9.77 ± 3.38 |
| IL-12α/IL-12β | 1.38 ± 1.39 | 0.3 ± 0.26 | 0.41 ± 0.36 |
| IL-16 | 2221.23 ± 405.21 | 2646.52 ± 706.27 | 2720.94 ± 863.72 |
| IL-17A | 1.47 ± 1.21 | 1.66 ± 0.85 | 2.22 ± 1.2 |
| IL-17F | 13.59 ± 8.9 | 32.23 ± 26.73 | 39.61 ± 30.76 |
| IL-1α | 130.76 ± 110.75 | 440.4 ± 396.38 | 384.01 ± 323.23 |
| IL-1β | 0.34 ± 0.22 | 0.49 ± 0.34 | 0.43 ± 0.23 |
| IL-2 | 1.32 ± 0.31 | 1.62 ± 0.49 | 1.48 ± 0.39 |
| IL-21 | 1.28 ± 1.11 | 0.86 ± 0.51 | 1.86 ± 1.05 |
| IL-22 | 16.35 ± 8.64 | 10.96 ± 11.36 | 6.6 ± 2.79 |
| IL-27 | 3.49 ± 1.64 | 8.3 ± 3.05 | 8.29 ± 3.66 |
| IL-3 | 0.04 ± 0.04 | 0.06 ± 0.08 | 0.08 ± 0.03 |
| IL-33 | 0.99 ± 1.09 | 1.6 ± 0.9 | 1.05 ± 0.86 |
| IL-4 | 0.14 ± 0.04 | 0.19 ± 0.12 | 0.18 ± 0.07 |
| IL-5 | 0.28 ± 0.12 | 0.47 ± 0.47 | 0.31 ± 0.07 |
| IL-6 | 11.87 ± 17.73 | 39.21 ± 51.36 | 40.82 ± 28.5 |
| IL-7 | 0.74 ± 0.39 | 0.93 ± 0.56 | 1.05 ± 0.48 |
| IL-9 | 1.75 ± 1.38 | 4.38 ± 8.57 | 1.11 ± 0.4 |
| PD-L2/PDCD1LG2 | 2264.68 ± 595.27 | 2364.98 ± 476.05 | 2396.86 ± 436.04 |
| TNF | 6.39 ± 3.29 | 13.75 ± 4.91 | 13.83 ± 4.93 |

Multiplexed quantification of proteins was performed in plasma samples collected t=27 weeks. Protein levels are expressed in Normalized Protein expression (NPX) units on a log2 scale. Data are shown as mean ± SD. Abbreviations: (CTLA-4) cytotoxic T-lymphocyte associated protein 4, (FGF21) fibroblast growth factor 2, (G-CSF/CSF3) granulocyte colony-stimulating factor, (GM-CSF/CSF2) granulocyte-macrophage colony-stimulating factor, (HGF) hepatocyte growth factor, (I-TAC/CXCL11) interferon-inducible T-cell alpha chemoattractant, (IFN) interferon, (IL) interleukin, (KC/CXCL1) keratinocyte-derived chemokine, (M-CSF/CSF1) macrophage colony-stimulating factor, (MCP-1/CCL2) monocyte chemoattractant protein 1, (MCP-5/CCL12) monocyte chemoattractant protein 5, (MDC/CCL22) macrophage-derived chemokine, (MIG/CXCL9) monokine induced by gamma interferon, (MIP-1β/CCL4) macrophage inflammatory protein 1β, (MIP-2/CXCL2) macrophage inflammatory protein 2, (PD-L1/CD274) programmed death-ligand 1, (PD-L2/PDCD1LG2) programmed death-ligand 2, (RANTES/CCL5) regulated on activation, normal T-cell expressed and secreted, (TARC/CCL17) thymus and activation-regulated chemokine, (TNF) tumor necrosis factor.

Text in the Methods (lines 204-213 in the revised manuscript with tracked changes): “**4.3 Olink Target 48 Mouse Panel Analysis.** Protein expression was analyzed using the Olink Target 48 Mouse Panel (Olink Proteomics, Uppsala, Sweden), which enables multiplexed quantification of 45 mouse proteins. Mouse plasma samples were prepared according to the manufacturer’s instructions. Briefly, 1 µL of each sample was incubated with paired oligonucleotide-labeled antibody probes. Upon binding to the target protein, the proximity of the probes enabled hybridization and extension by DNA polymerase, creating a unique PCR target sequence. Quantification was performed via high-throughput qPCR. Protein levels were expressed in Normalized Protein expression (NPX) units on a log2 scale as well was the absolute concentrations. Data quality control and normalization were carried out using Olink’s NPX Signature software (v2.0.2). Statistical analyses were performed using the NPX values. Proteins that were not detected are not shown.”

Text in the results (lines in 448-456 in the revised manuscript with tracked changes): “Proteomics further revealed elevated levels of several pro-inflammatory factors (e.g., MCP-1/CCL2, MIP-1β/CCL4, IL-1α, IL-3, IL-6, TNF), but also metabolic factors (e.g., fibroblast growth factor 2 (FGF21) and some anti-inflammatory factors (e.g., IL-10) in HFD+sham mice compared with Chow mice (Table 4). Notably, IL-22 levels were specifically decreased in HFD+sham mice. Furthermore, in HFD+WATx mice, IFN-ɣ levels were reduced while IL-17F concentrations were elevated compared with Chow mice. However, no significant differences in protein concentrations were observed between HFD+WATx and HFD+sham mice. Of note, eWAT lipectomy seemed to impact several cytokines and chemokines in a counterregulatory way based on numerical trends of their absolute concentrations compared with HFD+sham. Absolute concentrations (in ng/mL) of each protein are provided in S5 Table.”

Text in discussion (lines 682-705 in the revised manuscript with tracked changes): “eWAT lipectomy had no significant effect on inflammatory plasma proteins: Multiple inflammatory markers were increased in HFD+sham mice compared with Chow controls but there were no statistically significant differences between HFD+sham and HFD+WATx mice, albeit several cytokines and chemokines seemed to be impacted in a counterregulatory way based on the absolute concentrations. This may contradict results from others showing significant reductions in TNF-α and IL-1β levels in the circulation of eWAT-lipectomized mice [13]. This may be due to a difference in experimental conditions since lipectomy was performed on a chow diet (i.e., leaner mice), while the present study was performed in the context of a much more severe condition, HFD-induced obesity. Under the conditions employed herein many other metabolically dysfunctional and inflamed tissues (e.g., liver, vasculature, pancreas) as well as immune cells can contribute to the plasma pool of inflammatory factors, and potentially mask the effects of eWAT lipectomy. This may also explain why we did not observe significant effects on metabolic profile (e.g., body weight, plasma cholesterol and triglyceride levels) and metabolic comorbidities (e.g., liver disease, atherosclerosis). Since eWAT lipectomy in this study was only partial, the difference in secretory proteins released into the circulation might be therefore too small to detect with current techniques. Of note, although effects did not reach statistical significance regarding a specific cytokine or chemokine, it is still possible that the net effect of the many insignificant reductions of cytokines and chemokines together may impact the brain. Moreover, the sample size may have been insufficient to detect statistically significant differences. Increasing the sample size in future studies could help better characterize the impact of eWAT lipectomy on circulating inflammatory markers. Future studies should also consider to select specific cytokines and chemokines on basis of our findings allowing a more targeted (statistical) analysis, and to measure fluxes of WAT-derived cytokines with kinetic labeling, rather than their steady-state concentrations, for a more comprehensive understanding of systemic processes.”

In addition, we believe that other bioactive molecules than adipokines and cytokines, such as lipidic compounds, may also play a role in the effects of eWAT lipectomy on cerebrovascular function observed in the present study. We therefore additionally measured plasma free fatty acids (FFAs) as additional potential mediators. The results are displayed in Table 3. We observed a pronounced effect of eWAT lipectomy on specific polyunsaturated fatty acids (PUFAs): eWAT lipectomy significantly increased the plasma levels of eicosatrienoic acid (C20:3) and docosahexaenoic acid (C22:6, DHA) compared with sham-operated (HFD+sham) mice. In the case of DHA, the effect is particularly remarkable because Chow and HFD+sham had comparable levels, and DHA was specifically elevated in the HFD+WATx group. Eicosatrienoic acid and DHA are bioactive fatty acids, and both were previously shown to have anti-inflammatory properties [14,15]. Furthermore, in particular, DHA has been demonstrated to be beneficial neural membrane fluidity for cerebrovascular function in both mice and humans [16–18]. Overall, these findings show that eWAT lipectomy modulates the levels of specific FFAs in the circulation. The associated increase in eicosatrienoic acid and DHA may then, at least partly, explain the observed improvements in cerebrovascular function following eWAT lipectomy, although these changes are not sufficient to enhance memory function and learning abilities. For the information of the Editor: We used on purpose plasma collected at t=14 weeks (i.e., 1 month after surgery) because lipid-associated processes (e.g., exchange of lipids in cell membranes) are often slow processes (many weeks or months), and we are intrigued by the observation that lipectomy can have an effect on fatty acids with known beneficial effects. The analyses of the underlying mechanism is however probably very complex (e.g., several lipases and other factors do affect metabolic fluxes in feeding and fasting state) and beyond the scope of the present study.

New table 3:

Table 3: Concentrations of plasma FFAs measured at t=14 weeks (1 month after surgery).

| **Plasma FFAs (nmol/ml)** | **Chow** | **HFD+sham** | **HFD+WATx** |
| --- | --- | --- | --- |
| *Total SFAs* | *210.44* | *230.30* | *238.76* |
| **Lauric acid (12:0)** | **0.69^a^** | **0.82^b^** | **0.84^b^** |
| Myristic acid (14:0) | 8.14 | 8.58 | 8.44 |
| Pentadecanoic acid (15:0) | 4.83 | 6.29 | 5.30 |
| Palmitic acid (16:0) | 131.98 | 137.08 | 143.55 |
| **Margaric acid (17:0)** | **4.30^a^** | **5.25^b^** | **4.86^b^** |
| **Stearic acid (18:0)** | **54.09^a^** | **65.16^b^** | **68.68^b^** |
| Arachidic acid (20:0) | 2.81 | 2.90 | 2.98 |
| Behenic acid (22:0) | 2.24 | 2.56 | 2.58 |
| Lignoceric acid (24:0) | 1.36 | 1.66 | 1.52 |
| *Total MUFAs* | *174.78* | *194.94* | *210.25* |
| Myristoleic acid (14:1) | 2.54 | 3.09 | 2.90 |
| Palmitoleic acid (16:1) | 45.58 | 35.80 | 34.87 |
| **Oleic acid (18:1)** | **112.40^a^** | **142.37^b^** | **157.65^b^** |
| Eicosenoic acid (20:1) | 5.26 | 5.46 | 6.26 |
| Erucic acid (22:1) | 6.37 | 5.49 | 5.71 |
| Nervonic acid (24:1) | 2.63 | 2.74 | 2.86 |
| *Total PUFAs* | *163.15* | *145.92* | *164.79* |
| **Linoleic acid (18:2)** | **94.16^a^** | **64.27^b^** | **70.92^a,b^** |
| **α-linolenic acid (ALA, 18:3)** | **8.89^a^** | **5.76^b^** | **5.90^b^** |
| **Stearidonic acid (18:4)** | **0.52^a^** | **0.43^b^** | **0.43^b^** |
| **Eicosadienoic acid (20:2)** | **2.67^a^** | **3.75^b^** | **4.01^b^** |
| **Eicosatrienoic acid (20:3)** | **5.73^a^** | **7.61^b^** | **8.90^c^** |
| **Arachidonic acid (20:4)** | **24.66^a^** | **36.23^b^** | **43.77^b^** |
| Eicosapentaenoic acid (EPA, 20:5) | 4.84 | 4.00 | 4.22 |
| **Docosadienoic acid (22:2)** | **2.17^a^** | **1.66^b^** | **1.58^b^** |
| **Adrenic acid (22:4)** | **1.74^a^** | **2.68^b^** | **2.76^b^** |
| **Docosapentaenoic acid (DPA, 22:5)** | **4.54^a^** | **5.84^b^** | **5.87^b^** |
| **Docosahexaenoic acid (DHA, 22:6)** | **13.23^a^** | **13.68^a^** | **16.42^b^** |

Free fatty acid (FFA) concentrations were measured at t=14 weeks (1 month after surgery). Data are shown as mean ± SD. Statistical differences are indicated in bold; groups with the same superscript letters are statistically comparable (p>0.05) and groups with different superscript letters are statistically different (p≤0.05). Abbreviations: (MUFAs) monounsaturated fatty acids, (PUFAs) polyunsaturated fatty acids, (SFAs) saturated fatty acids.

Text in the Methods (line 201-203 in the revised manuscript with tracked changes): “In addition, free fatty acid (FFA) concentrations were determined in fasting plasma samples collected at t=14 weeks using a targeted lipidomics approach on LC-MS/MS system as previously described in detail [19].”

Text in the Results (lines 426-435 in the revised manuscript with tracked changes): “We first assessed whether eWAT lipectomy had effects on bioactive mediators in the circulation, including plasma FFAs, cytokines and chemokines. FFA concentrations were measured in plasma 1 month after surgery by lipidomics (Table 3). Concentrations of several FFAs including saturated fatty acids (e.g., lauric acid, margaric acid, stearic acid), monounsaturated fatty acids (e.g., oleic acid) and polyunsaturated fatty acids (PUFAs, e.g., eicosadienoic acid, eicosatrienoic acid, arachidonic acid, adrenic acid, docosapentaenoic acid) were increased upon HFD feeding relative to Chow. HFD feeding also triggered decreases in some PUFAs including α-linolenic acid, linoleic acid, stearidonic acid and docosadienoic acid. Interestingly, when compared to HFD+sham, eWAT lipectomy specifically increased the PUFAs eicosatrienoic acid and docosahexaenoic acid (DHA), i.e., fatty acids with beneficial anti-inflammatory properties.”

Text in the Discussion (lines 706-718 in the revised manuscript with tracked changes): “While eWAT lipectomy did not significantly affect cytokine concentrations in plasma, it did however modulate the levels of specific circulating FFAs. Following lipectomy, the levels of the PUFAs eicosatrienoic acid and DHA increased, both of which have been shown to possess anti-inflammatory properties [14,15,20]. Moreover, higher DHA levels have been suggested to have beneficial effects on brain blood perfusion and blood-brain barrier integrity [16,17]. Altogether, the observed elevations of FFAs with anti-inflammatory and vaso-protective properties may provide a mechanistic rationale for the observed effects of eWAT lipectomy on the brain. We cannot exclude that cytokines or chemokines are also mediating such effects because we employed an untargeted (48 panel) analysis which requires correction for multiple testing. Future lipectomy studies should include cytokine/chemokine measurements, because the numerical changes of plasma cytokine and chemokine concentrations suggest that lipectomy may counter-regulate the detrimental effects of HFD feeding, although statistical significance was not reached in the present study. Future studies evaluating the effect of WAT lipectomy could use our dataset to select cytokines/chemokines for more targeted analyses.”

**Comment from the editor:** 4. Reanalyze CBF data to explicitly compare pre- vs. post-surgery trends within groups, as the current interpretation of “masked aging effects” is speculative without direct statistical support.

**Response from the** **authors:** We apologize for this unclarity. We have performed the requested statistical comparisons, which demonstrate that cerebral blood flow (CBF) decreases over time (with aging) in all the groups in the hippocampus, and only in the Chow and HFD+WATx groups in the thalamus. We adapted the Methods, Results and Discussion accordingly.

Text in the Methods (lines 322-328 in the revised manuscript with tracked changes): “For CBF outcomes measured under normal gas mix, an additional repeated-measured GLM with Bonferroni correction was performed to assess CBF changes over time within the three experimental groups Chow, HFD+sham and HFD+WATx including t=7 weeks, t=12 weeks and t=26 weeks timepoints. For this specific analysis at t=7 weeks (prior to surgery), the combined HFD group was retrospectively divided into HFD+sham and HFD+WATx subgroups, mirroring the group separation used at the post-surgery time points.”

Text in the results (lines 501-509 in the revised manuscript with tracked changes): “CBF outcomes measured under normal gas mix were additionally analyzed over time between pre-surgery (t=7 weeks) and post-surgery time points (t=12 weeks and t=26 weeks) in Chow, HFD+sham and HFD+WATx groups, to further characterize the effect of aging on CBF. For this specific analysis at t=7 weeks (prior to surgery), the combined HFD group was retrospectively divided into HFD+sham and HFD+WATx subgroups, mirroring the group separation used at the post-surgery time points. No time effect was observed in the cortex (overall time effect: p=0.099). In the hippocampus, CBF significantly decreased over time in all the groups (overall time effect: p=0.012). In the thalamus, CBF also decreased over time in the Chow group (time effect: p=0.002) and the HFD+WATx group (time effect: p=0.001), while it remained stable in the HFD+sham group (time effect: p=0.715).”

Text in the Discussion (lines 719-730 in the revised manuscript with tracked changes): “In the present study, prolonged HFD feeding (>12 weeks) was associated with a higher CBF in comparison with mice fed a chow diet. In the thalamus especially, this HFD-induced increase in CBF seems to be due to a time-related decline in CBF in chow-fed mice, which is not observed in the HFD-fed mice. We therefore hypothesize that, under chow conditions, the process of aging tends to decrease CBF but, in obese (HFD) conditions, this decline is masked by a pathological increase of CBF that is associated with impaired vascular function of smaller vessels (e.g., capillaries) and larger vessels (e.g., atherosclerosis in aortic root).”

Text in the Discussion (lines 744-753 in the revised manuscript with tracked changes): “Interestingly, an age-related decline in CBF similar to chow-fed mice was observed in the thalamus of HFD-fed eWAT-lipectomized mice, suggesting that the eWAT lipectomy may attenuate the HFD-induced increase in CBF. In addition, partial eWAT lipectomy lowered hippocampal CBF under vasoconstrictive conditions (induced by pure oxygen) towards levels of the Chow group, while CBF remained higher in HFD+sham control group. This decrease in CBF under vasoconstrictive conditions indicates an amelioration of cerebral vasoreactivity in the hippocampus, which is consistent with previous research in patients with obesity showing associations between visceral WAT dysfunction and disturbed brain perfusion [21,22]. Our results are also in line with studies reporting beneficial effects of eWAT lipectomy on age-induced BBB permeability and brain damage in the context of ischemic brain injury [13].”

**Reviewers' comments:**

**Comment #1:** Please combine Figure 1 and Figure 2 in a picture and mark the words Ldlr-/- mice in Figure 1.

**Response from the authors:** We agree with the reviewer that combining Figure 1 and Figure 2 would be appropriate and adjusted this in the revised manuscript. The abbreviation Ldlr-/-.Leiden is also explained in the figure legend.

New Figure 1:

**Fig 1: Study design and general *in vivo* parameters.** (A) Study design: At t=0, 10-12 week-old low density lipoprotein receptor-deficient substrain ‘Leiden’ (Ldlr-/-.Leiden) male were either fed a high-fat diet (HFD group, n=34) or were kept on chow diet (Chow group, n=15) as reference control. At t=8 weeks, half of the HFD-treated mice underwent a sham surgery (HFD+sham group, n=17) and the other half underwent partial (~70%) eWAT lipectomy (HFD+WATx group, n=17). Cognitive performance was assessed at t=6 weeks (baseline; prior to surgery) with a Morris Water Maze (MWM) test, at t=11 weeks (i.e., 1 month after surgery) with an Object Recognition Test (ORT) and at t=26 weeks (i.e., 4 months after surgery) with a reverse MWM test. Systolic blood pressure (SBP) was measured at t=5 weeks and t=26 weeks. The mice also underwent brain magnetic resonance imaging (MRI) at t=7 weeks (prior to surgery), t=12 weeks (i.e., 1 month after surgery) and t=27 weeks (i.e., 4 months after surgery) to study cortical thickness, hippocampal volume, grey and white matter integrity as well as brain perfusion and resting-state functional connectivity. Animals were housed in digital ventilated cages and home-cage activity was measured 24 hours/day. eWAT samples were collected during partial eWAT lipectomy for *ex-vivo* culture (secretome analysis) and histology. At the end of the study (i.e., t=28 weeks), the (residual) eWAT, sWAT and mWAT depots of all mice were collected for *ex-vivo* culture and histology, and hippocampi were collected and used for RNAseq analyses to examine biological processes altered by HFD feeding and effects of partial eWAT lipectomy in the brain. (B) Food intake, (C) fat intake and (D) body weight were measured at t=5 weeks (prior to surgery), t=14 weeks (1 month after surgery) and t=27 weeks (4 months after surgery). At the same time points, (E) triglyceride, (F) cholesterol and (G) insulin concentrations were measured in 5-hour fasted plasma, and (H) blood glucose levels were measured in whole blood. (I) Systolic blood pressure (SBP) was assessed at t=5 weeks and t=26 weeks. Data are shown as mean ± SD. ↑ increase over time for all the groups (p ≤ 0.05), ↓ decrease over time for all the groups (p ≤ 0.05). * p ≤ 0.05, ** p ≤ 0.01, *** p ≤ 0.001 between the experimental groups.

**Comment #2:** In Figure S1, only statistical diagrams are available. Please add representative histological pictures.

**Response from the authors:** We agree with the reviewer that including representative images for each staining would better illustrate the results. We have incorporated these in the revised manuscript. We also did the same for Figure S2 (mWAT and sWAT histology) and Figure S5 (brain histopathology). Thank you for this comment to improve the manuscript.

New S1 Fig:

**S1 Fig: Liver disease and atherosclerosis at the end of the study**. (A) Representative pictures of liver and aortic root cross-sections respectively stained with hematoxylin-eosin (HE) and hematoxylin-phloxine-saffron (HPS). (B) Liver steatosis and (C) lobular inflammation were quantified in HE-stained liver cross-sections at t=28 weeks, and (D) liver fibrosis was measured as the percentage of Sirius red-positive area. Quantification of (E) The number of atherosclerotic lesions, (F) average lesion size and (G) total lesion size were quantified in HPS-stained cross-sections of aortic roots at t=28 weeks. Data are shown as mean ± SD. ** p ≤ 0.01, *** p ≤ 0.001.

New S2 Fig:

**S2 Fig: Histological analyses of mesenteric (mWAT) and subcutaneous (sWAT) WAT at the end of the study.** (A) Representative pictures of mWAT and sWAT histology at t=28 weeks. WAT weight, inflammation (crown-like structures, CLS) and adipocyte size were histologically analyzed in (B-D) mWAT (inguinal) and (E-G) sWAT. Data are shown as mean ± SD. ** p≤0.01, *** p ≤ 0.001.

New S5 Fig:

**S5 Fig: Histological markers of neuroinflammation, vascular integrity and neurogenesis at the end of the study.** Brain samples were analyzed histopathologically at t=28 weeks. (A) Representative pictures. Quantification of (B-C) IBA-1-positive area as a marker for microglia activation in grey and white matter regions, (D) GFAP-positive area and (E) intensity as a marker of astrogliosis, and (F-G) GLUT-1-positive area as an indicator of vascular integrity. (H) Neurogenesis was assessed by quantification of DCX-positive neurons in the dentate gyrus of the hippocampus. Data are shown as mean ± SD. Abbreviations: (DCX) doublecortin; (GFAP) glial fibrillary acidic protein; (GLUT-1) glucose transporter 1, (IBA-1) ionized calcium-binding adapter molecule 1.

**Comment #3:** What is the control group for these values in Table 1? Please explain the result.

**Response from the authors:** We thank the reviewer for the question and apologize for the lack of clarity. Table 1 details the molecules secreted by the portion of WAT tissue surgically removed from HFD-fed mice. We cultured the surgically removed eWAT to gain insight in the type and amounts of molecules that would be secreted into the tissue culture medium which is a synthetic medium that does not contain mouse proteins. Specifically, we removed a substantial portion of the eWAT tissue during lipectomy, intentionally leaving some of the depot intact. Unlike typical lipectomy studies where the removed tissue is discarded, we cultured the tissue *ex vivo*. By culturing the tissue in cell culture medium under the described conditions, we were able to estimate the secretory capacity of the removed portion. We measured the concentrations of cytokines and other inflammatory factors released into the medium, and compared them to control wells without eWAT (thus containing blank medium only). The molecules released within 4 hours accumulated in the medium, providing insights into the secretory capacity of the removed eWAT. Consequently, HFD+sham animals that underwent sham surgery retained the ability to secrete these molecules (because their eWAT-mass was not reduced), while the HFD+WATx group did not, as they lacked the (cultured) portion of eWAT. These results are thus rather descriptive in nature and provide an estimate on effects that may take place in HFD+sham mice (still containing the entire eWAT), and that may lack in HFD+WATx mice (as a consequence of the portion of eWAT being removed). We have clarified this in the Results section.

Text in the Results (lines 383-391 of the revised manuscript with tracked changes): “Tissue samples of the surgically removed eWAT were cultured *ex vivo* to gain insight in the type of inflammatory factors being released by the removed portion of adipose tissue. The amount of inflammatory mediators that were secreted during a 4-hour period of culturing were determined in the supernatant of cell culture medium. Culture medium from wells treated the same way but not containing eWAT served as ‘blank’ control samples. Obtained data were normalized to the total eWAT weight to gain insight into the 4-hour secretion capacity of the entire eWAT depot (Table 1). This analysis shows that, although the eWAT depot hardly contained CLS at the time of surgery, it already had the capacity to secrete multiple (pro-inflammatory) factors, and it suggests that, upon surgery, mice of the HFD+WATx group were less exposed to these factors derived from eWAT.”

**Comment #4:** There are Figure1-9 in this article. Please modify no more than 6 Figures, for example, Figure 4 and 9 can be placed in the supplementary data.

**Response from the authors:** We combined Figure 1 and 2 as suggested in the first comment of the reviewer, and moved Figure 4 and Figure 9 in the Supplementary material.

**Comment #5:** In Figure 9, only sequencing results were analyzed, and no validation experiments were conducted on USP22. the conclusion "Partial eWAT lipectomy specifically inactivates USP22 in the hippocampus" lacks rigor.

**Response from the authors:** We agree that the above conclusion that USP22 protein is inactivated by eWAT lipectomy is a bit overstated. We adapted the text accordingly.

Text in the Results (lines 627-633 in the revised manuscript with tracked changes): “Canonical pathways did not differ significantly between HFD+WATx and HFD+sham groups (all -log(p)<2), but on the level of upstream regulators, the enzyme ubiquitin specific peptidase 22 (USP22) was predicted to be significantly inactivated upon lipectomy in HFD+WATx mice compared with HFD+sham mice (-log(p)=3.6, z-score=-2.0) as detailed in S6C Fig and S9 Table. The role of USP22 in detrimental HFD-induced hippocampal changes is further supported by the observation that USP22 activation was predicted to be higher (trend) in HFD+sham compared with Chow (HFD+sham vs Chow mice (-log(p)=9.2, z-score=1.0).”

Text in the Discussion (lines 819-822 in the revised manuscript with tracked changes): “The upstream regulator analysis (i.e., analysis of key regulators that orchestrate and control biological processes based on the observed downstream gene transcripts) of hippocampal RNAseq data predicted a possible (yet specific) inactivation of USP22 upon eWAT lipectomy.”

**References**

1.	Gart E, van Duyvenvoorde W, Caspers MPM, van Trigt N, Snabel J, Menke A, et al. Intervention with isoleucine or valine corrects hyperinsulinemia and reduces intrahepatic diacylglycerols, liver steatosis, and inflammation in Ldlr−/−.Leiden mice with manifest obesity-associated NASH. FASEB J. 2022;36: 1–18. doi:10.1096/fj.202200111R

2.	Seidel F, Fluiter K, Kleemann R, Worms N, van Nieuwkoop A, Caspers MPM, et al. Ldlr-/-.Leiden mice develop neurodegeneration, age-dependent astrogliosis and obesity-induced changes in microglia immunophenotype which are partly reversed by complement component 5 neutralizing antibody. Front Cell Neurosci. 2023;17: 1–17. doi:10.3389/fncel.2023.1205261

3.	Chusyd DE, Wang D, Huffman DM, Nagy TR. Relationships between Rodent White Adipose Fat Pads and Human White Adipose Fat Depots. Front Nutr. 2016;3. doi:10.3389/fnut.2016.00010

4.	Severi I, Perugini J, Ruocco C, Coppi L, Pedretti S, Di Mercurio E, et al. Activation of a non-neuronal cholinergic system in visceral white adipose tissue of obese mice and humans. Mol Metab. 2024;79: 101862. doi:10.1016/j.molmet.2023.101862

5.	Börgeson E, Boucher J, Hagberg CE. Of mice and men: Pinpointing species differences in adipose tissue biology. Front Cell Dev Biol. 2022;10: 1–8. doi:10.3389/fcell.2022.1003118

6.	Franczyk MP, He M, Yoshino J. Removal of epididymal visceral adipose tissue prevents obesity-induced multi-organ insulin resistance in male mice. J Endocr Soc. 2021;5: 1–14. doi:10.1210/jendso/bvab024

7.	Mulder P, Morrison MC, Wielinga PY, Van Duyvenvoorde W, Kooistra T, Kleemann R. Surgical removal of inflamed epididymal white adipose tissue attenuates the development of non-alcoholic steatohepatitis in obesity. Int J Obes. 2016;40: 675–684. doi:10.1038/ijo.2015.226

8.	Jia Z, Wang Z, Pan H, Zhang J, Wang Q, Zhou C, et al. Crosstalk between fat tissue and muscle, brain, liver, and heart in obesity: cellular and molecular perspectives. Eur J Med Res. 2024;29: 637. doi:10.1186/s40001-024-02176-w

9.	Seidel F, Morrison MC, Arnoldussen I, Verweij V, Attema J, de Ruiter C, et al. Obesity accelerates age-related memory deficits and alters white matter tract integrity in Ldlr-/-.Leiden mice. Brain, Behav Immun - Heal. 2025;45: 100991. doi:10.1016/j.bbih.2025.100991

10.	Lee M, Lee KJ, Kim J, Lee DY, Park RW, Rhee SY, et al. Low-density lipoprotein cholesterol levels and risk of incident dementia: A distributed network analysis using common data models. J Neurol Neurosurg Psychiatry. 2025; 1–9. doi:10.1136/jnnp-2024-334708

11.	Baradaran H, Dahlstrom KA, Culleton S, Sarrami AH, McFarland MM, Romero JR. Association between Extracranial Carotid Artery Plaque and Cognitive Dysfunction: A Systematic Review and Meta-Analysis. Dement Geriatr Cogn Disord. 2022;51: 377–385. doi:10.1159/000526822

12.	Cheke LG, Bonnici HM, Clayton NS, Simons JS. Obesity and insulin resistance are associated with reduced activity in core memory regions of the brain. Neuropsychologia. 2017;96: 137–149. doi:10.1016/j.neuropsychologia.2017.01.013

13.	Shin JA, Jeong SI, Kim M, Yoon JC, Kim HS, Park EM. Visceral adipose tissue inflammation is associated with age-related brain changes and ischemic brain damage in aged mice. Brain Behav Immun. 2015;50: 221–231. doi:10.1016/j.bbi.2015.07.008

14.	Baker EJ, Miles EA, Calder PC. A review of the functional effects of pine nut oil, pinolenic acid and its derivative eicosatrienoic acid and their potential health benefits. Prog Lipid Res. 2021;82: 101097. doi:10.1016/j.plipres.2021.101097

15.	Banaszak M, Dobrzyńska M, Kawka A, Górna I, Woźniak D, Przysławski J, et al. Role of Omega-3 fatty acids eicosapentaenoic (EPA) and docosahexaenoic (DHA) as modulatory and anti-inflammatory agents in noncommunicable diet-related diseases – Reports from the last 10 years. Clin Nutr ESPEN. 2024;63: 240–258. doi:10.1016/j.clnesp.2024.06.053

16.	Hong S-H, Khoutorova L, Bazan NG, Belayev L. Docosahexaenoic acid improves behavior and attenuates blood–brain barrier injury induced by focal cerebral ischemia in rats. Exp Transl Stroke Med. 2015;7: 3. doi:10.1186/s13231-014-0012-0

17.	Patan MJ, Jackson PA. Shining a light on the effects of omega-3 polyunsaturated fatty acids on the brain: The relationship between cerebral blood flow parameters and cognition. Lipid Technol. 2016;28: 197–199. doi:10.1002/lite.201600052

18.	Weiser MJ, Butt CM, Mohajeri MH. Docosahexaenoic acid and cognition throughout the lifespan. Nutrients. 2016;8: 1–40. doi:10.3390/nu8020099

19.	Gart E, Salic K, Morrison MC, Caspers M, van Duyvenvoorde W, Heijnk M, et al. Krill oil treatment increases distinct pufas and oxylipins in adipose tissue and liver and attenuates obesity-associated inflammation via direct and indirect mechanisms. Nutrients. 2021;13. doi:10.3390/nu13082836

20.	Hsu L-C, Wen Z-H, Chen H-M, Lin H-T, Chiu C-M, Wu H-C. Evaluation of the Anti-Inflammatory Activities of 5,8,11-cis-Eicosatrienoic Acid. Food Nutr Sci. 2013;04: 113–119. doi:10.4236/fns.2013.49A1018

21.	Kiliaan AJ, Arnoldussen IAC, Gustafson DR. Adipokines: A link between obesity and dementia? Lancet Neurol. 2014;13: 913–923. doi:10.1016/S1474-4422(14)70085-7

22.	Vreeken D, Seidel F, de La Roij G, Vening W, den Hengst WA, Verschuren L, et al. Impact of White Adipose Tissue on Brain Structure, Perfusion, and Cognitive Function in Patients with Severe Obesity. Neurology. 2023;100: E703–E718. doi:10.1212/WNL.0000000000201538
